# ALS-Causing Mutations Significantly Perturb the Self-Assembly and Interaction with Nucleic Acid of the Intrinsically Disordered Prion-Like Domain of TDP-43

**DOI:** 10.1371/journal.pbio.1002338

**Published:** 2016-01-06

**Authors:** Liangzhong Lim, Yuanyuan Wei, Yimei Lu, Jianxing Song

**Affiliations:** 1 Department of Biological Sciences, Faculty of Science, National University of Singapore, Singapore; 2 NUS Graduate School for Integrative Sciences and Engineering, National University of Singapore, Singapore; Brandeis University, UNITED STATES

## Abstract

TAR-DNA-binding protein-43 (TDP-43) C-terminus encodes a prion-like domain widely presented in RNA-binding proteins, which functions to form dynamic oligomers and also, amazingly, hosts most amyotrophic lateral sclerosis (ALS)-causing mutations. Here, as facilitated by our previous discovery, by circular dichroism (CD), fluorescence and nuclear magnetic resonance (NMR) spectroscopy, we have successfully determined conformations, dynamics, and self-associations of the full-length prion-like domains of the wild type and three ALS-causing mutants (A315E, Q331K, and M337V) in both aqueous solutions and membrane environments. The study decodes the following: (1) The TDP-43 prion-like domain is intrinsically disordered only with some nascent secondary structures in aqueous solutions, but owns the capacity to assemble into dynamic oligomers rich in β-sheet structures. By contrast, despite having highly similar conformations, three mutants gained the ability to form amyloid oligomers. The wild type and three mutants all formed amyloid fibrils after incubation as imaged by electron microscopy. (2) The interaction with nucleic acid enhances the self-assembly for the wild type but triggers quick aggregation for three mutants. (3) A membrane-interacting subdomain has been identified over residues Met311-Gln343 indispensable for TDP-43 neurotoxicity, which transforms into a well-folded Ω-loop-helix structure in membrane environments. Furthermore, despite having very similar membrane-embedded conformations, three mutants will undergo further self-association in the membrane environment. Our study implies that the TDP-43 prion-like domain appears to have an energy landscape, which allows the assembly of the wild-type sequence into dynamic oligomers only under very limited condition sets, and ALS-causing point mutations are sufficient to remodel it to more favor the amyloid formation or irreversible aggregation, thus supporting the emerging view that the pathologic aggregation may occur via the exaggeration of functionally important assemblies. Furthermore, the coupled capacity of TDP-43 in aggregation and membrane interaction may critically account for its high neurotoxicity, and therefore its decoupling may represent a promising therapeutic strategy to treat TDP-43 causing neurodegenerative diseases.

## Abstract 2

The prion-like domain of TDP-43 appears to have an energy landscape that allows oligomerisation only under very limited conditions; however, TDP-43 mutations that cause amyotrophic lateral sclerosis are sufficient to remodel the protein in favor of amyloid formation.

## Author Summary

Amyotrophic lateral sclerosis (ALS) is the most prevalent fatal motor neuron disease. It was identified ~140 years ago, but the exact mechanism underlying the disease has still not been well defined. TAR-DNA-binding protein-43 (TDP-43) was identified as the major component of the proteinaceous inclusions present in ~97% ALS and ~45% frontotemporal dementia (FTD) patients, and has also been observed in an increasing spectrum of other neurodegenerative disorders, including Alzheimer disease. The TDP-43 C-terminus is a key domain—it encodes a prion-like domain and, crucially, hosts almost all ALS-causing mutations. Here we have successfully determined the conformations, dynamics, and self-associations of the prion-like domains of both wild type and three ALS-causing mutants in both aqueous solutions and membrane environments. The study suggests that the TDP-43 prion-like domain appears to have a unique energy landscape, which allows the assembly of the wild-type sequence into specific oligomers only under very limited conditions. Intriguingly, ALS-causing point mutations remodel the energy landscape to favor amyloid formation or irreversible aggregation, thus supporting the emerging view that pathologic aggregation may occur via the exaggeration of functionally important assemblies. Furthermore, the coupled capacity of TDP-43 in aggregation and membrane interaction may partly account for its high neurotoxicity; decoupling these may therefore represent a promising therapeutic strategy to treat TDP-43-mediated neurodegenerative diseases.

## Introduction

The TAR-DNA-binding protein-43 (TDP-43) was initially identified as a factor capable of binding to the TAR DNA of HIV and repressing transcription [1], which are well conserved among *Caenorhabditis elegans*, *Drosophila*, mouse, and human [2]. In 2006, the human TDP-43 was identified as the major constituent of the proteinaceous inclusions that are characteristic of most forms of amyotrophic lateral sclerosis (ALS) and the most common pathological subtype of frontotemporal dementia-frontotemporal lobar degeneration with TDP-43-positive inclusions (FTLD-TDP) [3,4]. TDP43 is an intrinsically aggregation-prone protein [3–14], and its irreversible aggregation has been found in ~97% ALS and ~45% FTD patients. Additionally, TDP-43 immunoreactive inclusions have also been observed in an increasing spectrum of other neurodegenerative disorders, which include ALS/parkinsonism–dementia complex of Guam, Alzheimer disease (AD), dementia with Lewy bodies (DLB), Pick’s disease, argyrophilic grain disease and corticobasal degeneration (reviewed in 5,12). Very recently, TDP‑43 has been identified as a key player in the clinical features associated with Alzheimer disease, in particular, cognitive impairment [15].

**Fig 1:**
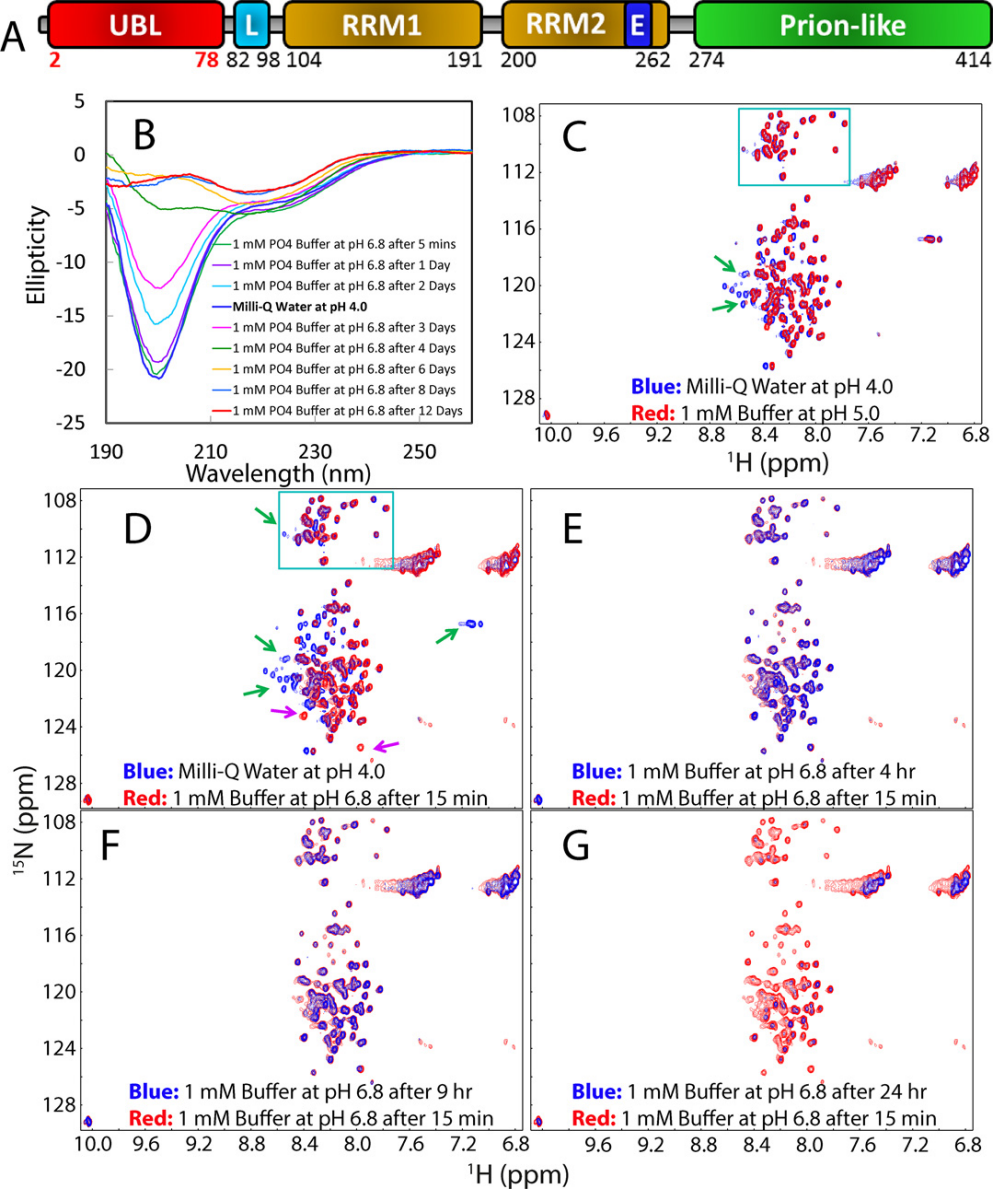
Characterization of the wild-type TDP-43 prion-like domain. (A) Domain organization of the 414-residue TDP-43 protein, which is composed of the N-ubiquitin-like domain, nuclear localization signal (L), two RNA recognition motifs (RRM1 and RRM2) hosting a nuclear export signal (E), and C-terminal prion-like domain abundant in Gln/Asn/Ser/Gly residues. (B) Far-UV CD spectra collected at 25°C for the prion-like domain over Lys263-Met414 at protein concentrations of 20 μM in Milli-Q water at pH 4.0 and in 1 mM phosphate buffer at pH 6.8 at different time points. (C) Superimposition of the two-dimensional NMR ^1^H-^15^N HSQC spectra of the prion-like domain acquired at 25°C, and at a protein concentration of 100 μM in Milli-Q water at pH 4.0 (blue), and in 1 mM phosphate buffer at pH 5.0 after 15 min (red). Green box is used to indicate the HSQC peaks of Gly residues while green arrows are used to indicate the HSQC peaks disappeared at pH 5.0. (D) Superimposition of HSQC spectra of the prion-like domain at 25°C in Milli-Q water at pH 4.0 (blue) and in 1 mM phosphate buffer at pH 6.8 after 15 min (red). Pink arrows are used to indicate the HSQC peaks manifested at pH 6.8. (E) Superimposition of HSQC spectra of the prion-like domain at 25°C in 1 mM phosphate buffer at pH 6.8 after 15 min (blue) and after 4 hr (red). (F) Superimposition of HSQC spectra of the prion-like domain at 25°C in 1 mM phosphate buffer at pH 6.8 after 15 min (blue) and after 9 hr (red). (G) Superimposition of HSQC spectra of the prion-like domain at 25°C in 1 mM phosphate buffer at pH 6.8 after 15 min (blue) and after 24 hr (red).

TDP-43 is a member of the heterogeneous nuclear ribonucleoprotein (hnRNP) family, which includes some of the well-known splicing modulators, such as hnRNP I, hnRNP A/B, and hnRNP H [5–7,12–14,16]. Previously, the 414-residue TDP43 was established to be composed of a nuclear localization signal (NLS), two RNA recognition motifs (RRM1 and RRM2) hosting a nuclear export signal (NES), and C-terminal Q/N/S/G-rich domain (Fig 1A). The NLS and NES regulate the shuttling of TDP-43 between the nucleus and the cytoplasm [17], while the RRM1 and RRM2 have been characterized to bind to a large variety of nucleic acids including single- or double-stranded DNA/RNA [17–21]. Intriguingly, the C-terminal domain over residues 274–414 has a low-complexity sequence abundant in Gln, Asn, Ser, and Gly residues, which shares 24.2% sequence identity with the N-terminal yeast prion domain of Sup35 [13,14,22]. The critical role of the C-terminus in ALS pathogenesis has also been strongly highlighted by the fact that this domain hosts almost all known ALS-associated mutations and has also been proposed to be responsible for the prion-like spreading of ALS [2,5–7,12–14], thus called prion-like domain [22–25]. Noticeably, such low-complexity domains have been shared by a large number of RNA-binding proteins, many of which were identified to be involved in neurodegenerative disease [12–14,22,25,26]. The functional studies revealed that the TDP-43 prion-like domain functions to form reversible oligomers or high-order granule by self-association or complexing with protein partners such as other hnRNPs [5–7,12–14,16,22,25–27]. For example, TDP-43 was shown to form 50–250 nm granule in the nucleus, providing a scaffold for other functionally related sub-compartments, that participates in transcriptional repression as well as alternative splicing [14,28]. On the other hand, in the cytoplasm, TDP-43 also participates in forming RNP granule, which include processing bodies (P-bodies) and stress granule (SGs) [5,13,29,30]. As such, it has been recently proposed that the physiological and reversible structures of TDP-43 serve as precursors, which transform into irreversible inclusions under certain pathological conditions [5,13,29,30].

Since its discovery, the mechanism for the TDP-43 aggregation has become a central focus, although the exact role of the aggregation in TDP-43 neurotoxicity remains controversial [31]. The TDP-43 oligomerization/aggregation appears to be cooperatively mediated by several regions, particularly by N- and C-termini which have very high tendency to aggregate in vitro and in vivo [31–33]. Surprisingly, recent studies revealed that like superoxide dismutase 1 (SOD1), TDP-43 is also capable of becoming associated with mitochondria to impair mitochondrial dynamics and function in motor neurons [34,35]. Previously, it has been extensively demonstrated that ALS-causing mutations transform the cytosolic SOD1 into a membrane-interacting protein that thus became associated with organelles such as mitochondria [36–38]. Indeed, recently the peptides derived from the TDP-43 prion-like domain were identified to have the membrane-damaging capacity [39].

Due to its extremely important role in a large spectrum of neurodegenerative diseases, the neurotoxicity of the wild-type and mutant TDP-43 have been extensively investigated by various cell and animal models [5–8,11–14,16 20, 26, 27, 31, 32, 34, 35,40–44]. On the other hand, the elucidation of the high-resolution structures, dynamics, and self-association of the TDP-43 domains, particularly for the prion-like domain, represents a crucial step towards delineating the mechanistic aspects of its physiological and pathological functions. Unfortunately, however, due to the intrinsic propensity of aggregation, this task has been significantly retarded except for the RRMs [21]. Consequently, only dissected prion-like fragments have been previously investigated by nuclear magnetic resonance (NMR) [43–45]. Very recently, as facilitated by our previous discovery that protein aggregation can be significantly minimized by reducing salt concentrations [46,47], we have successfully decrypted that the TDP-43 N-terminus unexpectedly encodes a novel ubiquitin-like fold coexisting with its unfolded form in equilibrium, thus rationalizing its high tendency in aggregation [33].

Here by circular dichroism (CD), fluorescence, electron microscopy, and NMR studies on the full-length prion-like domains of the wild type and three ALS-causing mutants, namely A315E, Q331K, and M337V, we aimed to gain insights into three important aspects associated with the TDP-43 proteinopathies: (1) What are the high-resolution pictures of the conformation, dynamics and self-assembly of the wild-type prion-like domain? (2) How do the ALS-causing mutations affect these properties? (3) Which unique feature might account for the high neurotoxicity of the TDP-43 inclusion? Our study reveals that although the TDP-43 prion-like domain is intrinsically disordered only with some nascent secondary structures, it has the capacity to assemble into dynamic oligomers by self-association or interacting with nucleic acid. Unexpectedly, all three ALS-causing point mutations are able to significantly perturb this capacity. Furthermore, we identified a region previously demonstrated to be indispensable for the TDP-43 neurotoxicity is in fact a membrane-interacting subdomain. Taken together, our results support the emerging view that the pathologic aggregation of proteins including TDP-43 in neurodegenerative diseases may occur via the exaggeration of functionally important and reversible assemblies [5,6,14,22,25,26,29,30,48–50]. Our study also implies that the coupling of the TDP-43 aggregation and membrane interaction might at least partly account for its high toxicity.

## Results

### 1. TDP-43 Prion-Like Domain Is Intrinsically Disordered but Starts to Self-Assemble at Neutral pH

The TDP-43 C-terminus over residues 263–414 containing the full-length prion-like domain (274–414) and a short linker (263–273) (Fig 1A) was cloned, expressed, and purified as described in Methods. It is highly soluble in Milli-Q water (pH 4.0) with a protein concentration up to 600 μM, similar to what we extensively found on other “insoluble” proteins, including the TDP-43 N-terminus [33,38,46,47]. As judged by its far-ultraviolet (UV) CD spectrum with the maximal negative signal at 199 nm and no positive signal at 190 nm (Fig 1B), it appears to be highly disordered without any stable secondary structure. Moreover, it has a ^1^H-^15^N heteronuclear single quantum coherence spectroscopy (HSQC) spectrum with very narrow ^1^H (0.86 ppm) and ^15^N (17.84 ppm) spectral dispersions in which three Trp residues have their sidechain HSQC peaks largely overlapped (Fig 1C). These observations indicate that it also has no tight tertiary packing. Nevertheless, the HSQC peaks are well separated, and the sample at 600 μM showed no detectable changes in CD and NMR spectra for several months in Milli-Q water (pH 4.0), thus allowing the collection of a large set of high-quality NMR spectra.

By diluting the protein in Milli-Q water into phosphate buffer, we were able to prepare the samples in 1 mM phosphate buffer at different pH values for CD and NMR characterization. In pH 5.0 buffer, the prion-like domain has a far-UV CD spectrum almost identical to that in pH 4.0 Milli-Q water, and an HSQC spectrum with the majority of peaks superimposable to those in pH 4.0 Milli-Q water, except for those of His-tag and several N-/C-terminal residues that become shifted or disappeared (Fig 1C). Evidently, HSQC peaks for Gly residues distributed over the whole sequence are almost completely superimposable under two conditions. Furthermore, the samples at 100 μM also showed no significant changes in CD and NMR spectra in pH 5.0 buffer for several weeks, suggesting that it has no significant conformational difference under two conditions, as well as no significant self-association.

We also characterized it in phosphate buffers at higher pH including 6.0 and 6.8. As shown in Fig 1B, the sample at pH 6.8 immediately prepared has a CD spectrum almost superimposable to that in Milli-Q water, implying that it also has no significant difference of secondary structures under two conditions. Consistent with CD results, except for the disappearance of peaks of the His-tag and some N-/C-terminal residues, many HSQC peaks including most of Gly residues are still superimposable under two conditions. This provides residue-specific evidence that the prion-like domain has no significant difference of the solution conformation at pH 4.0 and 6.8.

As temperature has large effects on the disordered proteins, we also systematically assessed the temperature-induced conformational changes at pH 4.0, 5.0 and 6.0 by CD spectroscopy (S1 Fig). Conformational changes are relatively small, in particular below 40°C. Therefore, in the present study we conducted all CD and NMR characterization at 25°C in aqueous solutions.

Interestingly, the sample, even at a concentration of 20 μM, started to self-associate in pH 6.8 buffer, as monitored by CD (Fig 1B). After 1 d, the CD signal intensity showed a slight reduction, while after 4 d, the CD spectrum changed dramatically which is similar to what have been observed on the soluble β-stranded oligomers formed by the peptides derived from the TDP-43 prion-like domain [43–45]. After 8 d, no further changes were detected and also no visible aggregate was formed. This implies that the wild-type prion-like domain is able to progressively assemble into larger but soluble oligomers at neutral pH. The deconvolution analysis of the CD spectra revealed that the soluble oligomer formed at 8 d contains ~3% helical but ~72% β-sheet and β-turn, as well as ~25% random coil conformations.

We also monitored the changes in pH 6.8 buffer by HSQC spectra at a protein concentration of 100 μM. At this higher protein concentration, the formation of larger oligomers appeared to be much faster. Even after 15 min, many HSQC peaks became broadened (Fig 1D), implying the occurrence of dynamic oligomerization. After 4 hr (Fig 1E) and 9 hr (Fig 1F), most peaks became very broad and consequently the intensity became weak. After 1 d, most peaks became too broad to be detectable (Fig 1G). Furthermore, the NMR sample in pH 6.8 buffer formed hydrogels after 1 d, which could change back to solution upon shaking, similar to what was also observed on the fused in sarcoma (FUS) prion-like domain [30,49]. However, higher protein concentrations such as at 200 μM would result in the rapid precipitation with white aggregates upon dilution into the buffer at pH 6.8.

**Fig 2:**
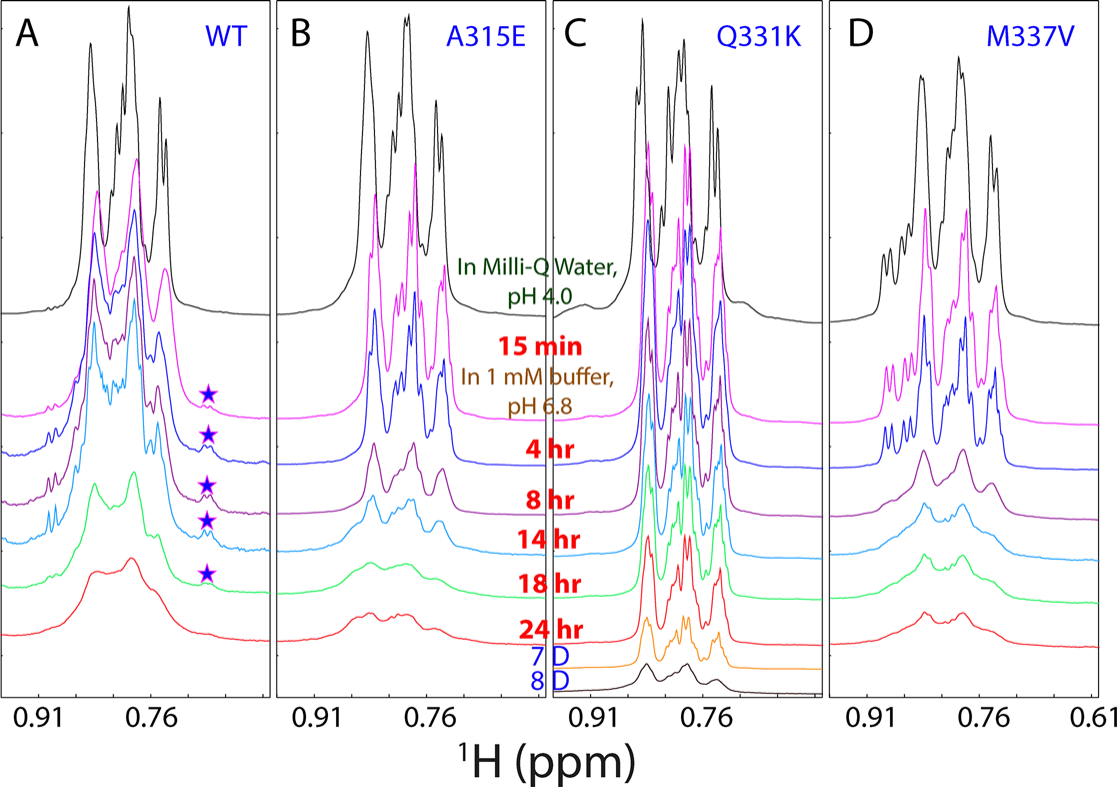
NMR characterization of the self-association. One-dimensional ^1^H NMR spectra over 0.6–0.96 ppm at different time points acquired at 25°C for the wild type (A), A315E (B), Q331K (C), and M337V (D), at a protein concentration of 40 μM in 1 mM phosphate buffer (pH 6.8). Stars are used to indicate the up-field NMR peaks manifested during the self-association only by the wild-type prion-like domain.

To slow down the self-association, particularly for the A315E and M337V mutants to allow detailed NMR characterization, we also acquired an array of one-dimensional ^1^H and HSQC spectra of the wild type and three mutants at a protein concentration of 40 μM in 1 mM phosphate buffer at pH 6.8. Fig 2 presents NMR spectra over 0.6–0.96 ppm, which are from the non-labile methyl protons. As seen in Fig 2A, in Milli-Q water at pH 4.0, there are only three large clusters of peaks and lacking of very up-field peaks, indicating that the wild-type prion domain is highly disordered, consistent with the above CD and NMR results. However, 15 min after the dilution of the wild type into 1 mM phosphate buffer at pH 6.8, two very up-field NMR peaks manifested respectively at 0.680 and 0.689 ppm (Fig 2A). At 14 hr, the intensity of the two peaks became the largest although the intensity of other peaks reduced significantly due to the self-association to form large oligomer. After 14 hr, the intensity of the two peaks started to reduce and mostly disappeared at 24 hr (Fig 2A). For proteins, such very up-field peaks are resulting from the methyl protons which have tight stack interaction with aromatic ring. As no new peaks manifested in the corresponding HSQC spectra, the manifestation of the up-field two peaks is most likely to result from the oligomeric form whose HSQC peaks were too broad to be detected. Interestingly, at a protein concentration of 40 μM in 1 mM phosphate buffer at pH 6.8, hydrogel was formed in NMR tube only after 1 week, much slower than that observed for the NMR sample at 100 μM.

### 2. ALS-Causing Mutants Have Very Similar Monomeric Conformations but Gain the Capacity to Form Amyloid Oligomers

To understand why some point mutations on the intrinsically disordered TDP-43 prion-domain are sufficient to trigger ALS, we successfully generated recombinant proteins of three ALS-causing mutants, A315E in the Ω-loop, Q331K in the middle of the helix, and M337V in the C-half of the helix (S2A Fig). As shown in S2B Fig, only Q331K has a CD spectrum slightly different from that of the wild type, whereas A315E and M337M have CD spectra almost superimposable to that of the wild type. The result clearly indicates that in aqueous solution, all three mutants are similarly disordered as the wild type. Indeed, except for the mutated residues, HSQC peaks of three mutants are also highly superimposable to those of the wild type. For example, the A315E mutant only has slight peak shifts of residues Gly314, Phe316, Ser317, and Ile318 which are close to the mutation site A315E in sequence (S2C Fig). On the other hand, the M337V mutant has slight peak shifts of residues Gly335 and Gly338 which are close to the mutation site, but of additional residues Leu340, Ser342 and Gln343 (S2E Fig). Interestingly, the Q331K mutant has slight peak shifts of the most extensive residues, which include Ala326, Gln327, Ala328, Ala329, Leu330, Ser333, Trp334, and Gly335, as well as Ala324 and Met337 (S2D Fig). Nevertheless, minor shifts of HSQC peaks of three mutants indicate that they have monomeric conformations very similar to that of the wild type, consistent with CD results.

**Fig 3:**
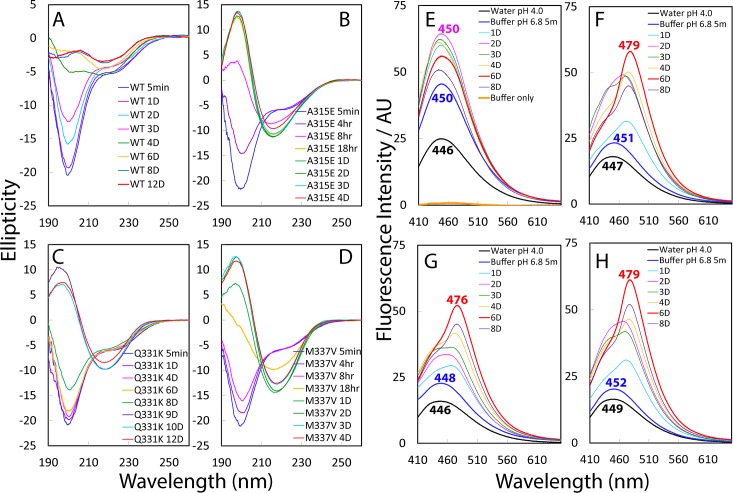
CD and fluorescence characterization of the self-association. Far-UV CD spectra acquired at 25°C at different time points of the incubation for the wild type (A), A315E (B), Q331K (C), and M337V (D) at a protein concentration of 20 μM in 1 mM phosphate buffer (pH 6.8). Emission spectra of the intrinsic visible fluorescence for the wild type (E), A315E (F), Q331K (G), and M337V (H) in water at pH 4.0, and in 1 mM phosphate buffer (pH 6.8) at different time points of the incubation. The wavelengths of the emission maxima are labeled for the spectra of the samples in water (pH 4.0), 5 min and 6 d after dilution into 1 mM phosphate buffer (pH 6.8). The wild type has an emission maximum very different from those of the three mutants.

We further conducted CD characterization of the self-association of three mutants at a protein concentration of 20 μM in 1 mM phosphate buffer at pH 6.8. Very unexpectedly, as shown in Fig 3A–3D, unlike the wild type, all three mutants finally transformed into the conformations which have CD spectra typical of the amyloid oligomers, as previously well-documented [43–45]. Interestingly, the A315E and M337V mutants transformed into the amyloid oligomers respectively after 18 hr (Fig 3B) and 1 d (Fig 3D), whereas it took 9 d for the Q331K mutant to complete the transformation (Fig 3C).

We also monitored the changes of NMR spectra over 0.6–0.96 ppm for three mutants together with the wild type under the exactly same conditions (Fig 2). Interestingly, three mutants showed no manifestation of two very up-field peaks during the self-association. Consistent with CD results (Fig 3), the A315E and M337V mutants largely completed the self-association at 8 hr (Fig 2B and 2D), while the Q331K mutant took 8 d (Fig 2C). Unlike the wild type, A315E and M337V mutants whose self-associations were significantly speeded up by higher protein concentrations used for NMR (40 μM), the amyloid-formation of Q331K is less concentration-dependent as it showed only a small difference at protein concentrations for CD (20 μM) and NMR (40 μM).

### 3. The Self-Association As Characterized by Three Fluorescence Probes

We further utilized fluorescence spectroscopy to characterize the self-association of the wild type and three mutants, which is one of the most common techniques to identify the formation of amyloid-like structures [51]. Briefly, we monitored the time-lapsed changes of three fluorescence probes, which include the intrinsic UV, visible fluorescence, and induced fluorescence by binding to Thioflavin T (ThT) [51–56].

The TDP-43 prion domain contains three Trp residues: Trp334, Trp385, and Trp412 and consequently has detectable intrinsic UV fluorescence [51]. Indeed, as shown in S3 Fig, the wild type and three mutants have very similar emission spectra in Milli-Q water (pH 4.0), with the emission maxima at ~351 nm, implying that three Trp residues have similar exposure in the wild type and three mutants. The slight intensity differences are most likely due to minor changes of the chemical environments triggered by mutations [51]. Interestingly, upon dilution into 1 mM phosphate buffer (pH 6.8), the wild type has the largest blue-shift of the emission maximum from 351 to 347 nm (S3A Fig), implying that the wild type started to self-assemble immediately, and consequently its Trp residues became more buried. After 1 d, the emission maximum of the wild type further blue-shifted to 342 nm, then to 339 nm after 6 d, and no more significant change occurred after 8 d (S3A Fig). Similar patterns of changes were observed for the three mutants. The results, particularly with the blue-shift of the emission maxima, suggest that the wild type and three mutants are all able to undergo the self-association into the oligomers in which Trp residues become more shielded from bulk solvent [51].

It is well established that the binding of ThT is a diagnostic probe for the formation of the β-rich amyloid structures although the exact molecular details still remain elusive [51]. So, we also monitored the binding of the wild type and three mutants to ThT at different incubation time points. Interestingly, as seen in S3E Fig, immediately upon dilution into the 1 mM phosphate buffer (pH 6.8), the wild type showed a large intensity of the ThT-binding induced fluorescence with the emission maximum at ~488 nm, implying its fast formation of the β-rich amyloid-like structure. After 1 d, the intensity was further increased and then reached the highest point after 2 d, followed by the reduction of the intensity afterward. The similar patterns were observed on A315E and M337V, but for Q331K, the intensity reached the highest point only after 4 d. The decrease of the ThT binding after long incubation times has been extensively observed because the fibrils may become packed together in such a way that the surface for ThT binding becomes less accessible [51,56].

Recently, it has been found that an intrinsic visible fluorescence develops during the β-rich fibrillar aggregation of amyloid-β (1–40) and (1–42), lysozyme as well as tau [54]. More specifically, this intrinsic visible fluorescence has been characterized to be independent of the presence of aromatic side-chain residues but to have its origin in the formation of special hydrogen bonds involved in the backbone C = O and N-H atom groups of peptide bonds, which already have electron delocalization to some degree. The formation of such hydrogen bonds will further enhance electron delocalization and thus allow low energy electronic transitions [53–55]. As shown in Fig 3E, the incubation buffer had no detectable visible fluorescence. However, even in water at pH 4.0, this intrinsic visible fluorescence could be detected for the wild type and three mutants even at 40 μM concentration, which have similar emission maxima: 446 nm for the wild type (Fig 3E), 447 nm for A315E (Fig 3F), 446 nm for Q331K (Fig 3G), and 449 nm for M337V (Fig 3H). This implies that for the TDP-43 prion-like domain, a large number of hydrogen bonds involved in backbone atoms already exist even in the highly-disordered monomeric states, because previously this visible fluorescence only became detectable for lysozyme (emission maximum at 425 nm) and γ-crystallin (emission maximum at 465 nm) in solution at a very high concentration: 100 mg/ml [53].

Remarkably, only 5 min after the dilution of the wild type into 1 mM phosphate buffer (pH 6.8), the intensity of this fluorescence was almost doubled (from reading of 24 to 45), with a slight red-shift of the emission maximum from 446 to 450 nm (Fig 3E). After 2 d, this fluorescence reached the highest with no significant shift of the emission maximum, followed by the reduction of the intensity afterwards. By a sharp contrast, the changes of this fluorescence showed a different pattern for three mutants. Upon immediate dilution into 1 mM phosphate buffer (pH 6.8), three mutants only showed slight intensity increases with slight red-shifts of the emission maxima (Fig 3F–3H). Nevertheless, after 1 d, the emission spectra of three mutants became very different from that of the wild type. For example, after 1 d, the emission maxima have significantly red-shifted to 475 nm for A315E (Fig 3F), 468 nm for Q331K (Fig 3G), and 472 nm for M337V (Fig 3H). Only after 6 d, the intensities of all three mutants reached the highest, with the emission maxima significantly red-shifted as compared to that for the wild type (450 nm): 479 nm for A315E, 476 nm for Q331K, and 479 nm for M337V.

The red-shift of the emission maximum of this intrinsic visible fluorescence has been established to be correlated to the amount and arrangement of the hydrogen bonds involved in the backbone peptide bonds: the higher the amount of hydrogen bonds arranged in the β-rich amyloid-like structures, the larger the red-shift will be [52–56]. For example, lysozyme with a mixture of α-helix and β-sheet secondary structures has the emission maximum at 425 nm, while γ-crystallin with β-sheet dominant secondary structures has its emission maximum significantly red-shifted to 465 nm [53]. Remarkably, the emission maxima for three mutants are even larger than that of an amyloid nanofibrils formed by Poly(ValGlyGlyLeuGly) peptide (~468 nm). Strikingly, this nanofibril has been found to become electricity-conductive due to its well-formed amyloid β-structures, which thus owns a highly ordered hydrogen bond network so as to allow radical electron delocalization [55].

Therefore, it appears that even in the highly disordered states of the wild type and three mutants in water at pH 4.0, there already exist a large amount of hydrogen bonds involved in the peptide bonds, as evidenced by their detectable intrinsic visible fluorescence. Furthermore, upon dilution into the buffer at pH 6.8, the prion-like domains of the wild type and three mutants will start the self-association, which thus leads to the formation of inter-molecular β-sheet structures, and consequently results in significant intensity increases of this fluorescence. Together with CD and NMR results, the results with this visible fluorescence suggest that the wild type self-assembles into the oligomer very different from those by three mutants. It appears that the oligomer formed by the wild type may still contain a small portion of disordered/dynamic regions lacking of hydrogen bonds which are involved in peptide bond atoms and arranged in β-amyloid structures. Consequently, the oligomer formed by the wild type has the emission maximum at 450 nm, larger than that of lysozyme (425 nm) but smaller than that of γ-crystallin (465 nm). By contrast, the three mutants, although upon immediate dilution they behave similarly to the wild type, acquired the capacity to further transform into the well-formed amyloid structures with very different CD spectra and emission maxima of this fluorescence (Fig 3). In the future, it is of fundamental interest to investigate whether the fibrils formed by the TDP-43 prion like domains, particularly by three mutants, are also conductive, and if yes, whether it has any relevance to the physiological functions or/and pathological roles.

### 4. Electron Microscopy Imaging

**Fig 4:**
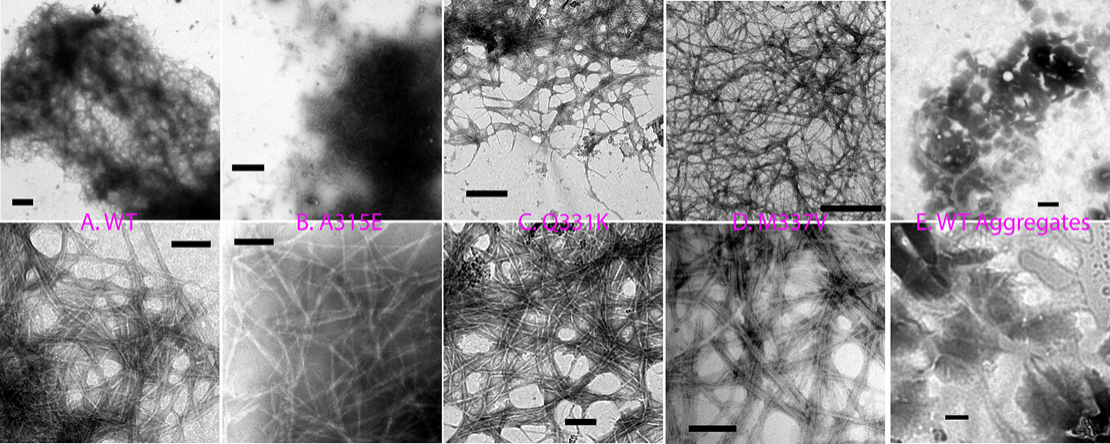
Electron microscope imaging. EM images of the samples incubated for 1 week for the wild type (A), A315E (B), Q331K (C), or M337V (D). (E) EM images of the aggregates rapidly formed by the wild type at a high protein concentration (200 μM). Upper row is images of lower magnification (scale bar of 1 μm) while lower row is of higher magnification (scale bar of 200 nm).

We further used electron microscope (EM) to visualize the morphology of the self-association for the wild type and three mutants at the incubation times of one and two weeks. As seen in Fig 4A–4D, after one week, the wild type and three mutants were all able to form amyloid fibrillar structures, with the widths of fibrils ranging from 15 to 30 nm, similar to the fine structures that were detected in the neuronal TDP-43 inclusions in patients’ brain tissues [57]. After 2 wk, the amyloid fibrillar structures remained similar but they appeared to become slightly more clustered/condensed together (S4 Fig). On the other hand, amorphous structures of diverse sizes were observed for the aggregates rapidly formed by diluting the wild type into 1 mM phosphate buffer at pH 6.8 to reach a concentration of 200 μM (Fig 4E). It is interesting to point out that the fibrillar structures in patients’ brain tissues have been demonstrated to fail to significantly bind ThT, thus representing non-classical amyloid fibrils. In the future, it is of fundamental interest to investigate whether the inability of the pathological TDP-43 aggregates to bind ThT might be due to the loss of accessibility of the ThT-binding sites as we observed here after long incubation times (S3E and S3H Fig).

### 5. The Residue-Specific Conformation and Dynamics in Aqueous Solution

**Fig 5:**
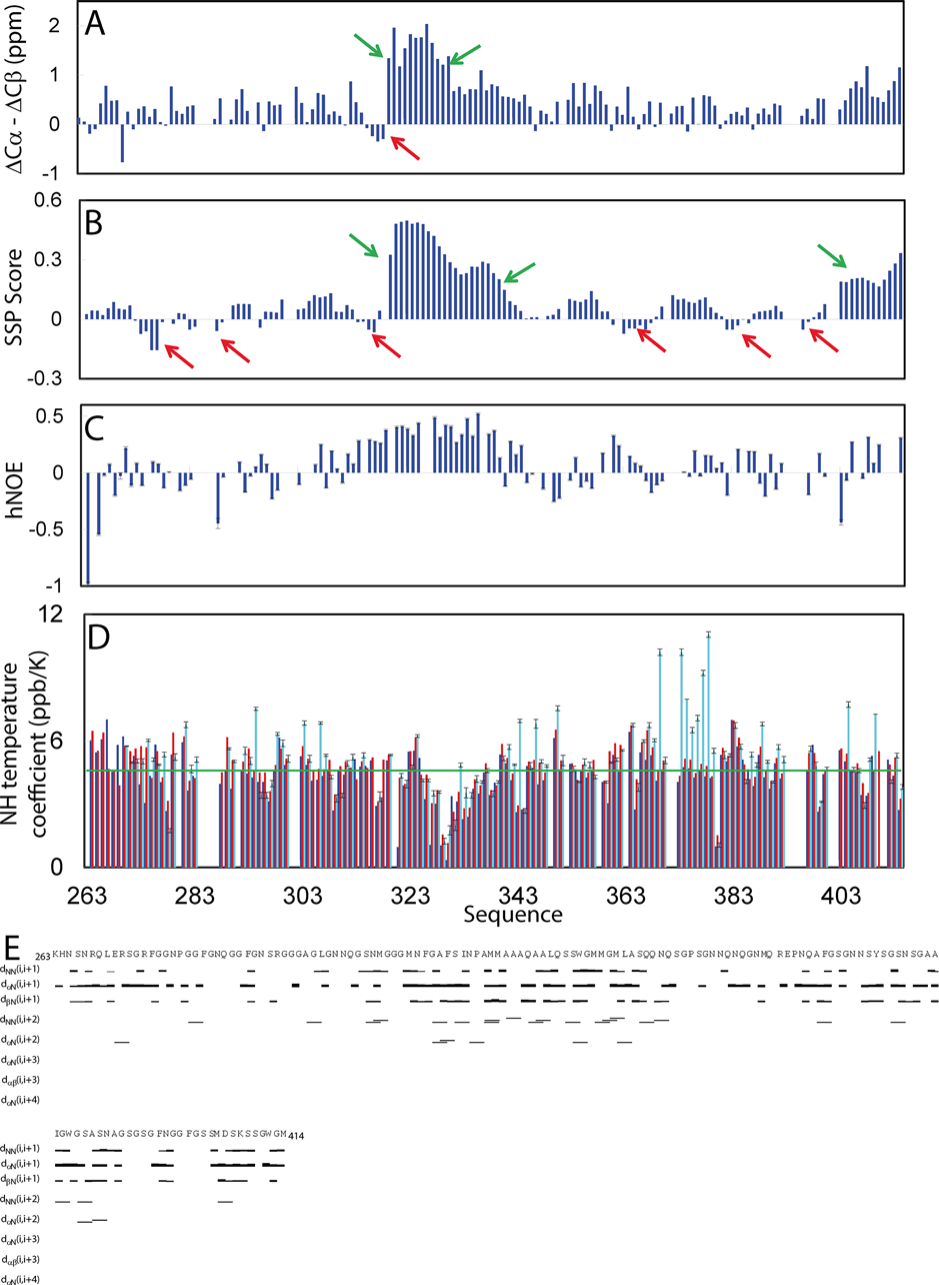
Residue-specific conformation and dynamics of the wild type prion-like domain in aqueous solution. (A) Residue specific (ΔCα-ΔCβ) chemical shifts of the prion-like domain at 25°C. Green arrows are used for indicating the regions populated with nascent helical conformations while red arrows for those with extended conformations. (B) Secondary structure score obtained by analyzing chemical shifts of the prion-like domain with the SSP program. A score of +1 is for the well-formed helix while a score of -1 for the well-formed extended strand. (C) {^1^H}-^15^N heteronuclear steady-state NOE (hNOE) of the prion-like domain at 25°C. (D) Residue-specific temperature coefficients of the wild-type prion-like domain in Milli-Q water at pH 4.0 (blue), in 1 mM phosphate buffer at pH 5.0 (red) and in 1 mM phosphate buffer at pH 6.0 (cyan). (E) NOE connectivities defining secondary structures of the prion-like domain. NMR data for preparing the above figures are presented in S1 Data.

Here, by analyzing three-dimensional NMR spectra including CCC(CO)NH, HN(CO)CACB, HSQC-TOCSY (total correlation spectroscopy), and HSQC-NOESY (nuclear Overhauser effect spectroscopy), we have successfully achieved the sequential assignments of all non-Proline residues except for residues Gln286-Gly287, Gly290-Asn291, Gly295, Asn301-Asn302, Asn371-Asn372, Ser393-Ser395, and Phe401-Gly402, whose NMR resonances were either undetectable or severely overlapped. Fig 5A presents the (ΔCα-ΔCβ) chemical shifts, which represent a sensitive indicator of the residual secondary structures in disordered proteins [38,58,59]. The small absolute values of (ΔCα-ΔCβ) chemical shifts over the whole sequence clearly indicate that it is indeed lacking of any stable secondary structure, completely consistent with its CD results (Fig 1B). Nevertheless, several regions have relatively large deviations. For example, residues Pro320-Gln331 all have the (ΔCα-ΔCβ) larger than 1 ppm, suggesting that this region is populated with helical conformation to some degree. Furthermore, despite very small, residues Ser317-Ile318-Asn319 have negative (ΔCα-ΔCβ) values, implying that they may adopt a relatively extended conformation, consistent with a previous finding that these residues were involved in forming an intermolecular β-sheet in the isolated peptide Met307-Asn319 [45].

To gain quantitative insights into the populations of different secondary structures, we further analyzed NH, N, Hα, Cα and Cβ chemical shifts of the prion-like domain by SSP program [60]. As seen in Fig 5B, all residues have the absolute values of SSP scores less than 0.5, confirming that the whole domain has no stable secondary structure. Nevertheless, residues Pro320-Leu340 have SSP scores larger than 0.2 while residues Ser403-Met414 have SSP scores larger than 0.15, thus implying that they are populated with helical conformations to some degree, resembling nascent helix. Indeed, residues Pro320-Leu340 were previously found to adopt stable helical conformation in an isolated peptide of Met311-Gln360 [43]. However, a detailed comparison with our present results is impossible as the previous NMR data and structure was not deposited. Interestingly, some short segments also have negative SSP scores, which include but are not limited to Gly274-Gly277, Gly314-Ser317, Pro363-Gly368, Ala382-Gly384, implying that these regions might have intrinsic capacity to adopt extended conformations to some degree. They may serve as seeds/nucleation sites to form amyloids or/and inclusions which are rich in extended β-conformations.

We also assessed the backbone rigidity by collecting heteronuclear NOEs, which provides a measure to the backbone flexibility on the pico- to nanosecond (ps-ns) timescale [33,38,58]. As shown in Fig 5C, the backbone is overall flexible on ps-ns time scale as judged from the small or even negative heteronuclear NOEs (hNOEs), with an average value of only 0.07 (Fig 5C). However, residues Gly314-Met339 have hNOE all larger than 0.25, implying that this region has relatively-restricted backbone motions, which is likely due to the presence of partly-populated secondary structures. Furthermore, analysis of the HSQC-NOESY spectrum indicated that even for Met311-Ala341, only some d_αN(i, i+2)_ and d_NN(i, i+2)_, but no d_αN(i, i+3)_ NOEs could be found (Fig 5E), suggesting that the helical conformations over this region are only dynamically populated in the full-length domain. The lack of stable secondary structure appears due to the abundance in polar residues Gln, Asn, Ser, and Gly in the TDP-43 prion-like domain. Indeed, all residues have negative hydrophobicity score [61], except for two regions Met311-Met339 and Ile383-Ser387 (S5A Fig).

### 6. Mechanism of the Self-Assembly

An interesting question is why the TDP-43 prion-like domain showed no significant oligomerization in water at pH 4.0, or even in 1 mM phosphate buffer at pH 5.0, but started to oligomerize at pH 6.8 although the initial solution conformations are very similar at these pH values? On the other hand, it is also interesting to observe very minor shifts of HSQC peaks at different pH values (Fig 1C and 1D), because for disordered proteins, their amide protons are expected to be highly exposed to bulk solvent. As a consequence, even minor changes of solution conditions such as pH will trigger significant shifts of HSQC peaks due to the changes of the chemical environments, although the conformation may remain largely unchanged [58], as we previously observed on the disordered ALS-causing SOD1 mutant [38], P56S-MSP domain [62] and isolated dengue NS3 protease domain [63]. This observation, together with the above results with the intrinsic visible fluorescence, strongly implies that the majority of the backbone amide protons might be involved in hydrogen bonding in the TDP-43 prion-like domain.

Therefore, we measured NMR temperature coefficients of the backbone amide protons in Milli-Q water at pH 4.0, in 1 mM phosphate buffers at pH 5.0 and pH 6.0 (Fig 5D), which represents a sensitive NMR probe for the involvement of the amide protons in hydrogen bonding [58,59,64]. Surprisingly, at pH 4.0, the backbone amide protons of the most residues have very small temperature coefficients, with an average of 4.3, which are not only much smaller than those of the disordered peptides [58,59] but also around or even smaller than 4.6 ppb/K, which was defined as an indicator of the involvement in well-formed hydrogen-bonds based on the studies on 793 backbone amides derived from 14 well-folded proteins [64]. This implies that the majority of the backbone amide protons are engaged in hydrogen bonding at pH 4.0. Previously, it has been found that the side chain atoms of Asn, Gln and Ser have particularly strong capacity in forming hydrogen bonds with the backbone atoms in both well-folded proteins as well as short peptides, which include the hydrogen bonds between the side chain oxygen and backbone amide protons [65,66]. One the other hand, it has been proposed that poly-Q sequences behave as polar zippers, which are able to oligomerize by forming inter-molecular hydrogen bonds between side-chain and backbone atoms [22,67]. As such, here we propose that in the TDP-43 prion-like domain at pH 4.0, most backbone amide protons are involved in forming hydrogen bond networks with the side-chain oxygen atoms, thus resulting in the very small NMR temperature coefficients and minor shifts of HSQC peaks at different pH, as well as manifestation of the intrinsic visible fluorescence (Fig 3E). These hydrogen bond networks might be most likely to be intra-molecular, rather than inter-molecular, as we have also collected CPMG-based ^15^N relaxation data on the TDP-43 prion-like domain as we previously did on SOD1 [38] but found no response, thus implying no significant inter-molecular association as protein association occurs on the μs-ms time scale, which should be detected by CPMG-based dispersion experiments [68]. In particular, this successfully rationalizes the above observation that the wild type and three mutants could have detectable intrinsic visible fluorescence even at a very low concentration (40 μM) in the water (pH 4.0), in which the TDP-43 prion-like domains are mostly monomeric and highly disordered (Fig 3E–3H).

On the other hand, at pH 5.0, most backbone amide protons have slightly larger temperature coefficients, with an average of 4.7 (Fig 5D). Furthermore, at pH 6.0, for the detectable HSQC peaks, they have further increased temperature coefficients (with an average of 5.2), in particular over Ser369-Asn378. These observations might be explained by the possibility that at higher pH, the exchange rates of most backbone amide protons increase due to the base-catalyzed exchange, as the prion-like domain is highly disordered with backbone amide protons largely accessible to bulk solvent [69]. As a result, at higher pH, the dissociation between amide proton and nitrogen atoms would become increased, thus leading to the disruption of the intra-molecular hydrogen-bonds involved in backbone amide protons of the TDP-43 prion-like domain at pH 4.0. As a consequence, at neutral pH, the side chains of Asn, Gln, and Ser will be mostly liberated and become available to form intermolecular polar zipper [22,67], or/and steric zipper [70].

We also determined the temperature coefficients of three mutants at different pH values (S6 Fig). However, for the A315E and M337V mutants, a large portion of HSQC peaks became too broad or even disappeared at pH 6.0 when temperature was above 30°C. As such, we were unable to obtain the temperature coefficients of the A315E and M337V mutants at pH 6.0. As shown in S6A and S6B Fig, most A315E residues have the temperature coefficients (with an average of 4.8 at pH 4.0 and 4.9 at pH 5.0, respectively) larger than those of the wild type. Similarly, most M337V residues (S6F and S6G Fig) also have the temperature coefficients (with an average of 4.9 at pH 4.0 and 5.0 at pH 5.0, respectively) larger than those of the wild type. By contrast, many Q331K residues have the temperature coefficients (with an average of 4.2 at pH 4.0, 4.6 at pH 5.0 and 4.9 at pH 6.0, respectively) slightly smaller than those of the wild type. This set of results thus implies that the incubation time needed for completing the self-association appears to be correlated to, but the conformations of the final self-associated states have no clear correlation to the overall stability of the inter-molecular hydrogen-bonding network, because the Q331K mutant has slightly lower average temperature coefficients than the wild type, but still transforms into the amyloid oligomer after long incubation time as reported by its CD (Fig 3C) and fluorescence (Fig 3G) spectra.

### 7. Interactions with Nucleic Acid by the Wild Type and Three Mutants

The low-complexity sequences homologous to the TDP-43 prion-like domain have been extensively identified in DNA/RNA-binding proteins, which function to form oligomers for binding a large spectrum of nucleic acids including single- and double-stranded DNA/RNA. Remarkably, a large set of such domains alone without RNA-binding motifs, which include those from FUS and TDP-43, have been recently characterized to be sufficient to bind nucleic acids to facilitate the assembly into dynamic β-dominant oligomers [30,49]. Therefore, here we used CD and NMR spectroscopy to characterize the interactions of nucleic acid with the wild-type and three mutant TDP-43 prion-like domains. As RNA triggered rapid self-associations or even aggregation, and thus did not allow the differentiation between the wild type and the mutants, here we used single-stranded DNA (ssDNA) which has been identified to bind TDP-43 [33]. To minimize the nucleic-acid-independent self-association at neutral pH as we showed above, we conducted the binding characterization in 1 mM phosphate buffer at pH 5.0, in which both wild-type and mutant TDP-43 prion-like domains showed no significant self-associations in one week.

Indeed, as characterized by CD spectroscopy, ssDNA was able to bind and trigger the significant conformational changes for the wild-type prion-like domain (S7A Fig). Interestingly, no aggregate was formed during the titration and the CD sample formed hydrogels even at pH 5.0 and a protein concentration of 20 μM shortly after reaching the ratio of 1:1, thus preventing from further adding ssDNA. By contrast, all three mutants showed the behaviors different from that of the wild type upon titrations with ssDNA. Once the ratio exceeded 0.3 for A315E (S7B Fig), 0.6 for Q331K (S7C Fig) and 0.4 for M337V (S7D Fig), the mutant proteins precipitated immediately with white aggregates and thus no good-quality CD spectra could be acquired further.

**Fig 6:**
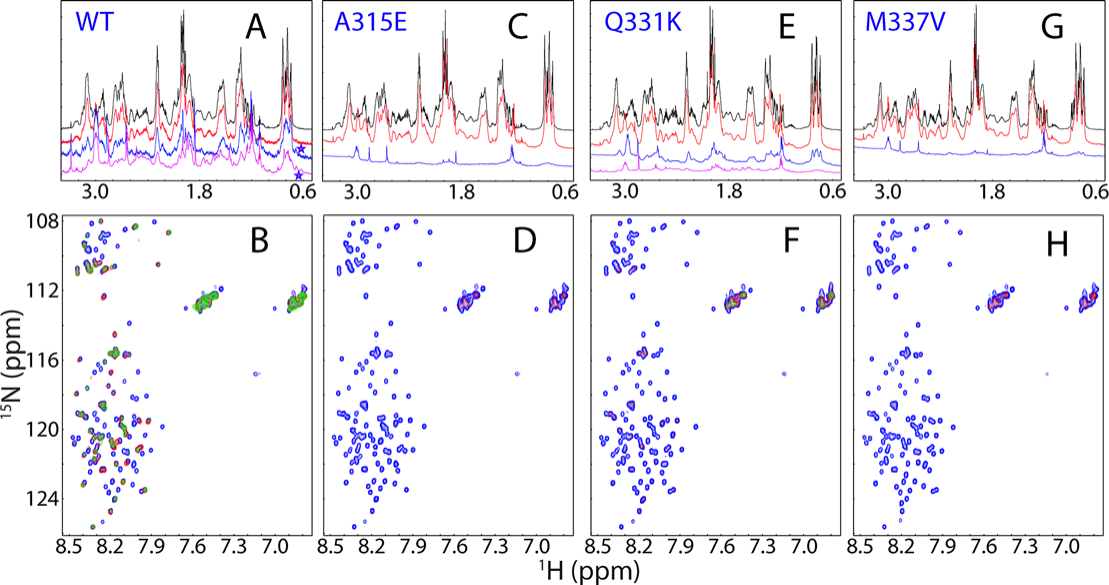
NMR characterization of the interactions with ssDNA. One-dimensional ^1^H NMR spectra over 0.5–3.4 ppm at molar ratios (protein:ssDNA) of 1:0 (black), 1:0.2 (red), 1:0.5 (blue) and 1:1 (pink), as well as HSQC spectra at molar ratios of 1:0 (blue), 1:0.5 (blue), and 1:1 (green), respectively, acquired at 25°C for the wild type: (A)–(B); A315E: (C)–(D); Q331K: (E)–(F); and M337V: (G)–(H) at a protein concentration of 40 μM in 1 mM phosphate buffer (pH 5.0). Star is used to indicate the up-field NMR peaks manifested upon interacting with ssDNA only by the wild type.

We also monitored the interaction of ssDNA with the wild-type and mutant prion-like domains by one-dimensional ^1^H NMR and HSQC spectra (Fig 6). For the wild type, the dilution of the protein into 1 mM phosphate buffer at pH 5.0 would not initiate the self-association as observed at pH 6.8 (Fig 2A), as evidently from the absence of any very up-field two NMR peaks (Fig 6A). However, upon addition of ssDNA at a ratio of 1:0.5 (protein:ssDNA), two very up-field peaks started to manifest and their intensity become much higher at a ratio of 1:1 (Fig 6A). Interestingly, the two peaks induced by ssDNA at pH 5.0 have chemical shifts (at 0.683 and 0.690 ppm respectively) similar to those of the oligomer (at 0.681 and 0.689 ppm respectively) formed at pH 6.8 without ssDNA. Furthermore, we also monitored the interactions by HSQC spectra (Fig 6B). Very interestingly, for the wild type, although at a ratio of 1:0.5 many peaks became too broad to be detected mostly due to the involvement in forming the large oligomer, a set of HSQC peaks is still detectable and mostly superimposable to those in the free state (Fig 6B). Based on the sequential assignment, the remaining peaks were all from the C-terminal residues Gln343-Met414 except for those from Gly304 and Gly309 (S8 Fig). This implies that the C-terminal residues Gln343-Met414 still remain largely flexible even in the oligomer of the wild-type prion-like domain complexed with ssDNA.

By contrast, all three mutants behaved very different from the wild type in interacting with ssDNA, as evidenced firstly by the absence of the very up-field peaks for A315E (Fig 6C), Q331K (Fig 6E), and M337V (Fig 6G). Secondly, upon exceeding certain ratios of protein:ssDNA, the mutant proteins precipitated rapidly with white aggregates and their 1D and HSQC peaks became too weak to be detected (Fig 6C–6H), consistent with CD results.

### 8. TDP-43 Prion-Like Domain Contains a Membrane-Interacting Subdomain

The capacity in disrupting the plasma and organelle membranes has been identified in all aggregation prone proteins causing human diseases including amyloid-β peptide [71–73], the diabetes related peptide IAPP [73–75] and SOD1 mutants [36–38]. Very recently, imaging studies revealed that mutant huntingtin inclusions appeared to be “engulfed” in the nuclear membrane to lead to eventual neuronal death [76]. Indeed, membrane-interacting fragments/domains have been identified in these proteins and their NMR structures have been determined in membrane environments [36,71–75,77,78], such as in lipid-mimetic dodecylphosphocholine (DPC) micelle, which is amenable to liquid NMR characterization at atomic resolution.

**Fig 7:**
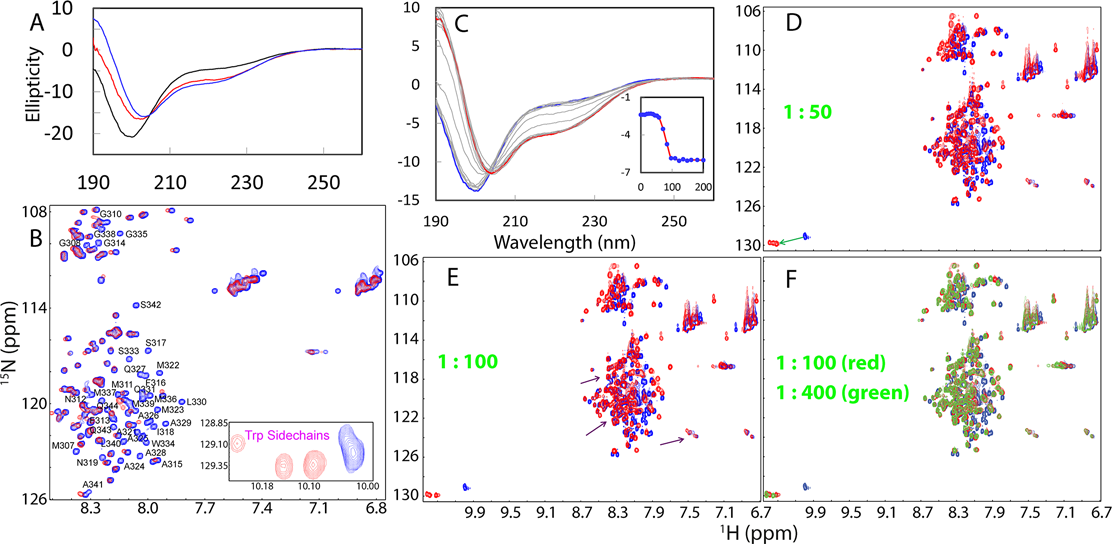
Interactions of the wild-type prion-like domain with membranes. (A) Far-UV CD spectra of the wild-type prion-like domain acquired at 25°C in Milli-Q water at pH 4.0 (black), in the presence of the DMPC/DHPC bicelle (red) and DPC micelle (blue) at a ratio of 1:200. (B) Superimposition of HSQC spectra of the prion-like domain acquired at 25°C in aqueous solution (blue) and in the presence of the DMPC/DHPC bicelle at a ratio of 1:200 (red). The assignments of the disappeared HSQC peaks are labeled. Inlet: HSQC peaks of three Trp side chains in aqueous solution (blue) and in the presence of DMPC/DHPC bicelle at a ratio of 1:200 (red). (C) Far-UV CD spectra of the prion-like domain acquired at 25°C in the presence of DPC micelle (blue) at different ratios (0–200). (D) Superimposition of HSQC spectra of the prion-like domain acquired at 25°C in aqueous solution (blue) and in the presence of DPC micelle at a ratio of 1:50 (red). (E) Superimposition of HSQC spectra of the prion-like domain acquired at 25°C in aqueous solution (blue) and in the presence of DPC micelle at a ratio of 1:100 (red). (F) Superimposition of HSQC spectra of the prion-like domain acquired at 25°C in aqueous solution (blue), in the presence of DPC micelle at a ratio of 1:100 (red) and 1:400 (green).

Here, we addressed the question of whether the TDP-43 prion-like domain also contains any membrane-interacting subdomain. To achieve this, we first titrated the prion-like domain with the large bicelle composed of DMPC/DHPC at a q value of 4, which resemble native bilayer membranes [79]. As seen in Fig 7A, in the presence of bicelle at a ratio of 1:200 (prion:bicelle), the prion-like domain has a far-UV CD spectrum very different from that in aqueous solution, with the maximal negative signal shifted from 198 to 203 nm; and a new negative signal at 222 nm, implying that the prion-like domain indeed has the subdomain which can interact with membranes to form helical conformation. Furthermore, in the presence of bicelle, many HSQC peaks underwent shifts and some even completely disappeared, as well as the peaks of three Trp side chains became well-separated (Fig 7B). This observation suggests that upon interacting with bicelle, each of Trp side chains has different chemical environment. Strikingly, the disappeared peaks were identified to be from residues Met307-Gln344, indicating that these residues became tightly associated with the large bicelle, consequently their HSQC peaks became too broad to be detected due to shortening of their T2 values [79].

We thus conducted further NMR characterization in DPC micelle which has much smaller size than bicelle, thus allowing the collection a large set of high-quality three-dimensional NMR spectra for determining the structures and dynamics [38,71–75,77–79]. We titrated the prion-like domain with DPC as monitored by CD and NMR HSQC. As judged from CD spectra in the presence of DPC at different ratios (Fig 7C), gradual addition of DPC induces progressive increase of the helical conformation, with the significant transition occurring over the ratios 50–100 (prion:DPC). Interestingly the CD spectra are very similar in DMPC/DHPC bicelle and DPC micelle (Fig 7A), suggesting the secondary structures of the prion-like domain are highly similar in two membrane environments. Furthermore, consistent with the CD results, HSQC titrations also showed that the prion-like domain underwent conformational changes with gradual addition of DPC, as indicated by the significant shifts of some HSQC peaks (Fig 7D and 7E). However, above the ratio of 1:100, the HSQC spectra showed only minor changes (Fig 7F). Similar to what is observed in bicelle, the HSQC peaks of three Trp side chains also becomes well-separated in DPC micelle. It is also interesting to note that many HSQC peaks in the presence of DPC even at a ratio of 1:400 (prion:DPC) are still superimposable to those in aqueous solution (Fig 7E and 7F). This clearly indicates that very different from what was observed on the SOD1 and P56S-MSP mutants that a large portion of residues became embedded in the membrane environment [38,78]. For the TDP-43 prion-like domain, only a small portion of the residues became tightly associated with DPC micelle.

**Fig 8:**
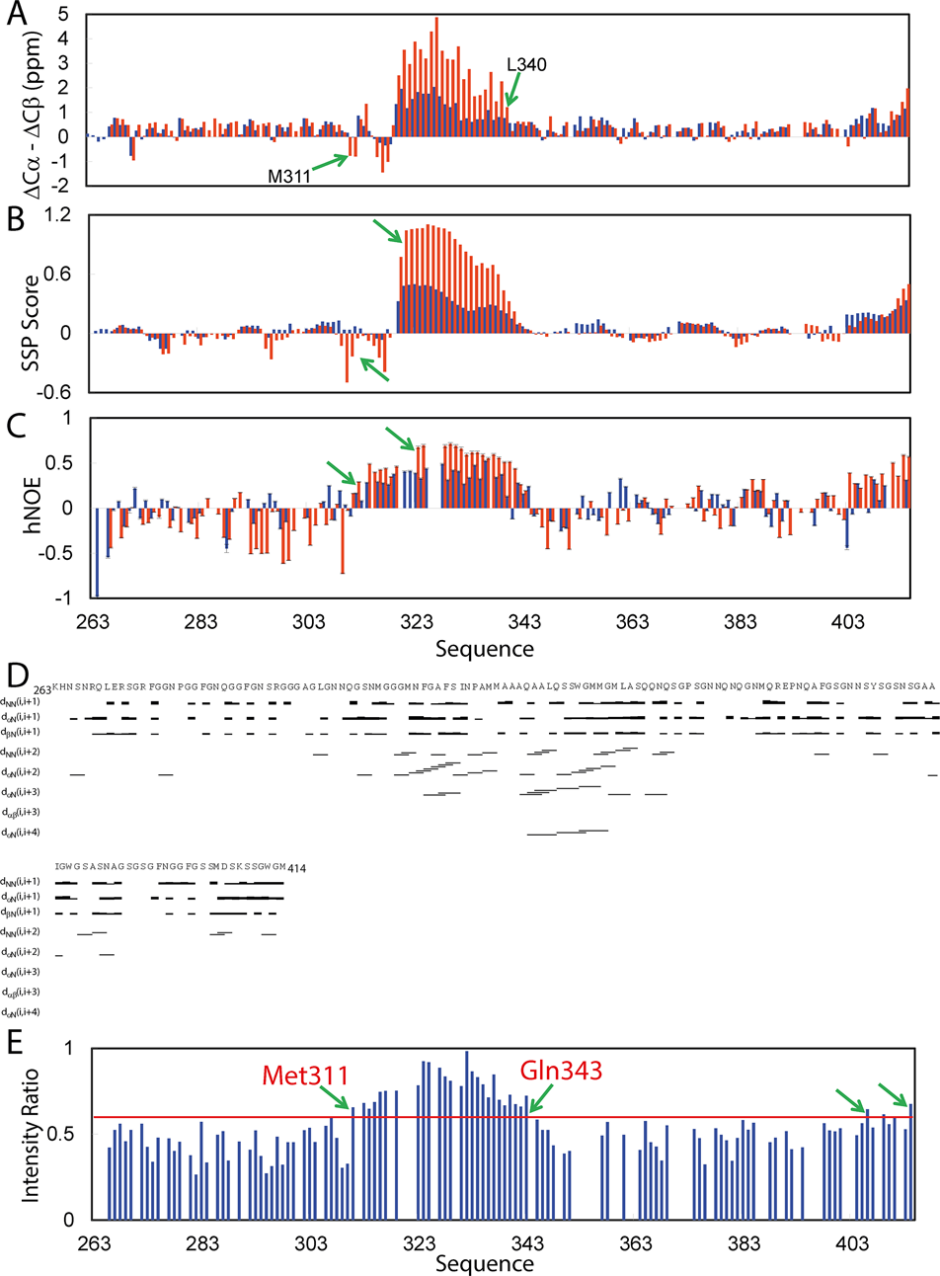
Residue-specific conformation of the wild-type prion-like domain in DPC micelle. (A) Residue specific (ΔCα-ΔCβ) chemical shifts of the prion-like domain in DPC micelle (red) and in aqueous solution (blue). (B) Secondary structure scores of the prion-like domain in DPC micelle (red) and in aqueous solution (blue), which were obtained by analyzing their chemical shifts of the prion-like domain with the SSP program. (C) {^1^H}-^15^N heteronuclear steady-state NOE (hNOE) of the prion-like domain in DPC micelle (red) and in aqueous solution (blue). (D) NOE connectivities defining secondary structures of the prion-like domain in DPC micelle. (E) Ratios of HSQC peak intensities of the prion-like domain in DPC micelle with and without 10 mM gadodiamide. Red line (0.59) representing the average value plus a standard deviation is set up as a cut-off. NMR data for preparing the above figures are presented in S2 Data.

We also successfully achieved sequential assignments of all non-Proline residues in the presence of DPC at a ratio of 1:200 (prion:DPC), except for residues Lys263-Ser266, Asn352, Asn371, Ser393-Gly394, and Gly402. Fig 8A presents its (ΔCα-ΔCβ) chemical shifts. Interestingly, in DPC micelle, residues Met311-Leu340 have (ΔCα-ΔCβ) chemical shifts significantly different from those in aqueous solution. More specifically, the (ΔCα-ΔCβ) chemical shifts of residues Met311-Asn312 and Phe316-Ile318 become more negative, implying that they become more extended. By contrast, the (ΔCα-ΔCβ) chemical shifts of residues Asn319-Leu340 become much more positive, strongly indicating that this region becomes highly helical. Indeed, SSP score analysis (Fig 8B) revealed that in DPC micelle, residues Gly309-Ile318 adopt more extended conformation while Asn319-Leu340 become well-formed helical conformation. In particular, residues Ala321-Gln331 all have SSP score larger than 0.9. The results together reveal that the residues Met311-Leu340 constitute the main region for interacting with both bicelle and DPC micelle. The C-terminal residues Trp442-Gly413-Met414 have slight increase in the helical conformation (Fig 8B), implying that these residues may transiently interact with DPC.

We collected CD spectra of the prion-like domain in DPC micelle at a ratio of 1:200 (prion:DPC) in Milli-Q water (pH 4.0), 1 mM phosphate buffers (pH 5.0 and 6.8), which are almost superimposable (S9A Fig). This result implies that the DPC-embedded conformations have no significant difference at three solution conditions. We also collected their HSQC spectra, and the results showed that at pH 5.0, most HSQC peaks remain superimposable to those at pH 4.0, only with some HSQC peaks disappeared which are from His-tag residues and several N-/C-terminal residues (S9B Fig). At pH 6.8, more peaks shifted and disappeared, but those from Gly310-Gln343 remain almost unperturbed (S9C Fig), implying that the backbone NH protons of these residues are not accessible to the bulk solvent.

**Fig 9:**
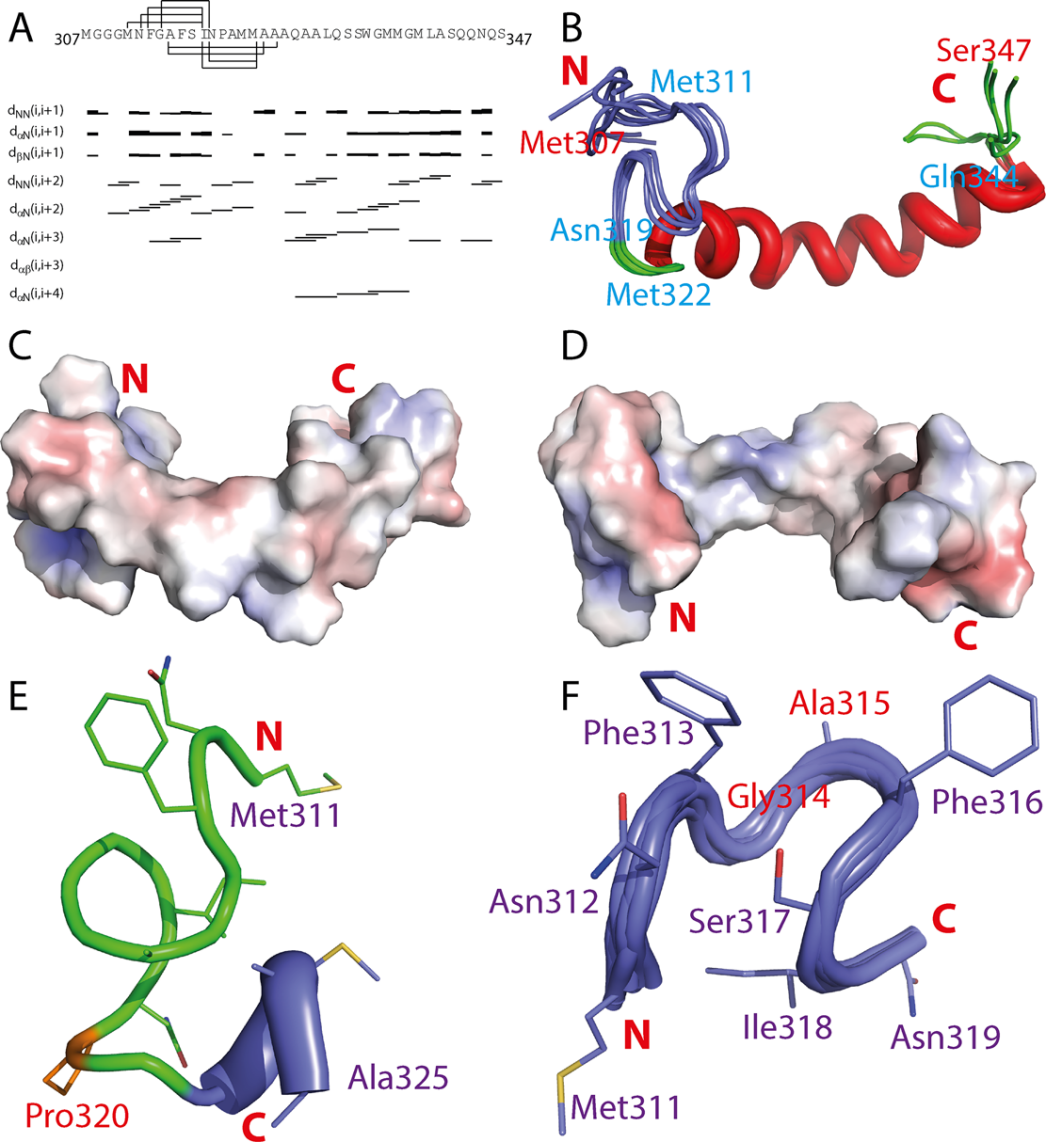
Three-dimensional structure of the membrane-interacting subdomain. (A) The sequence of the membrane-interacting subdomain Met307-Ser347 with assigned NOEs displayed. (B) Superimposition of six lowest energy structures of the membrane-interacting subdomain. (C)–(D) The electrostatic potential surfaces of the membrane-interacting subdomain structure with different orientations. (E) Structure of residues Met311-Ala325 with long-range NOEs adopting the Ω-loop and the N-part helix. (F) Superimposition of six lowest energy structures over the Ω-loop (Met311-Asn319) with the side chains of the lowest-energy structure displayed and labelled.

We collected heteronuclear NOEs in DPC micelle (Fig 8C). Consistent with chemical shift changes (Fig 8A), residues Met311-Ala341 in the presence of DPC have significantly more positive hNOEs than in aqueous solution, with an average of 0.53 (0.3 in aqueous solution). Additionally, the C-terminal residues Ser403, Asp406, Gly411-Met414 also have increased hNOEs. It is also intriguing to note that in DPC micelle, some residues such as Ser292-Gly309 have more negative hNOEs, implying that they become more flexible upon interaction with DPC. Further analysis of the HSQC-NOESY spectrum indicated that many d_αN(i, i+2)_ and d_NN(i, i+2)_, as well as d_αN(i, i+3)_ and d_αN(i, i+4)_ NOEs manifested in DPC micelle (Fig 8D). In particular, a large amount of NOEs were found over Gly309-Gln344. Surprisingly, 9 long-range NOEs were found over segment Met311-Ala325 (Fig 9A).

We explored the solvent accessibility of the residues of the prion-like domain in the membrane environment by titrations with a paramagnetic agent gadolinium(III) 5,8-bis(carboxylatomethyl)-2-[2-(methylamino)-2-oxoethyl]-10-oxo-2,5,8,11-tetraazadodecane-1-carboxylate hydrate), or gadodiamide, as we previously performed on the DPC-embedded SOD1 [38] and major sperm protein (MSP) mutants [78]. Gadodiamide with the paramagnetic gadolinium(III) coordinated has a large molecular volume and thus is only accessible to the protein atoms exposed to the bulk solvent. Upon titration to 10 mM, except for those of residue Met311-Gln343 and of several C-terminal residues, HSQC peaks of other residues have the intensity ratios less than the average value plus a standard deviation (0.59) (Fig 8E), implying that the amide protons of these residues are mostly exposed to the bulk solvent while those of Met311-Gln343 and several C-terminal residues are embedded in the membrane environment, thus inaccessible to gadodiamide [38,78].

### 9. Three-Dimensional Structure of the Membrane-Interacting Subdomain

SSP analysis and manifestation of a large amount of NOEs indicate that the membrane-interacting subdomain transforms into a well-folded structure in the membrane environment. Therefore, 50 NMR structures of the TDP-43 Met307-Ser347 were calculated by CYANA software package [38,80], with 328 NOE-derived distance and 46 TALOS-based dihedral angle [81] restraints (S1 Table). Six structures with the lowest target functions were selected for further refinement with AMBER force field [38,82] and the calculation statistics and structure quality are summarized in S1 Table.

Fig 9B presents the superimposition of six lowest-energy NMR structures, which are very similar with RMS deviations of 1.11 Å and 0.35 Å respectively for all and backbone atoms of the residues Met311-Gln344 (S1 Table). Fig 9C and 9D show the electrostatic surfaces of the lowest-energy structure. Due to the location of the hydrophobic side chains on the surface of the structure, the majority of the surface is highly hydrophobic (Fig 9C and 9D). While residues Met322-Gln344 adopt well-defined α-helix structure, residues Met311-Asn319 assume a well-defined but irregular loop structure (Fig 9E and 9F), which can be classified into Ω-loop [83–86]. Interestingly, completely different from a cytosolic Ω-loop of ephrin-B2 determined in aqueous solution, in which the hydrophobic sidechains formed a relatively buried core [86], in the TDP-43 Ω-loop formed upon being embedded in the membrane environment, the hydrophobic sidechains of Met311, Phe313, Ala315, Phe316, and Ile318 are pointed out to constitute a hydrophobic surface. This membrane-induce exposure of the hydrophobic side chains may play a key role in interacting with hydrophobic phase of membranes (Fig 9C–9F). In fact, the TDP-43 Ω-loop is similar to that of the human prothrombin gamma-carboxyglutamic acid-rich (GLA) domain which is required to anchor clotting proteins onto membrane surfaces in order to increase their local concentration for effective clotting [84,85]. In the Ω-loop of the GLA domain, three hydrophobic side chains are also highly exposed, which have been demonstrated to be absolutely essential for penetrating into the phospholipid bilayer [84,85].

Remarkably, the TDP-43 sequence forming the Ω-loop appears to be extremely critical for manifesting its neurotoxicity. Previously, a cellular model of the TDP-43 aggregation using the sequence 331–369 (lacking of the Ω-loop but containing the C-half of the helix) repeated 12 times has been established but surprisingly its aggregation is not toxic *per se* but instead has a protective role [31]. On the other hand, residues Met311-Asn319 exactly forming the Ω-loop (Fig 9F) has been identified as the minimal region to manifest neurotoxicity [44]. Furthermore, the peptide Met307-Met322 covering the Ω-loop was identified to be capable of disrupting liposomes but amazingly the deletion of Met307-Met311 completely eliminated the membrane-damaging capacity of the peptide Asn312-Met322 [39].

### 10. Conformations of the Three Mutants in Membrane Environments

To characterize the conformations of three ALS-causing mutants in membrane environments, we first titrated them in 1 mM phosphate buffer at pH 5.0 with DPC, and S10A Fig presents their CD spectra in the presence of DPC at a molar ratio of 1:200 (protein:DPC). Interestingly, although three mutation sites are located on the membrane-embedded subdomain, they have CD spectra very similar to that of the wild type, thus implying that the conformations of three mutant prion-like domains are very similar to that of the wild type in DPC micelle. Indeed, a further examination of their HSQC spectra shows that except for the mutation residue, the residues of three mutants, namely A315E (S10B Fig), Q331K (S10C Fig) and M337V (S10D Fig), have their HSQC peaks largely superimposable to those of the wild type. Moreover, in DPC micelle, the CD and HSQC spectra of the wild type and three mutants have no significant changes even after 1 month.

**Fig 10:**
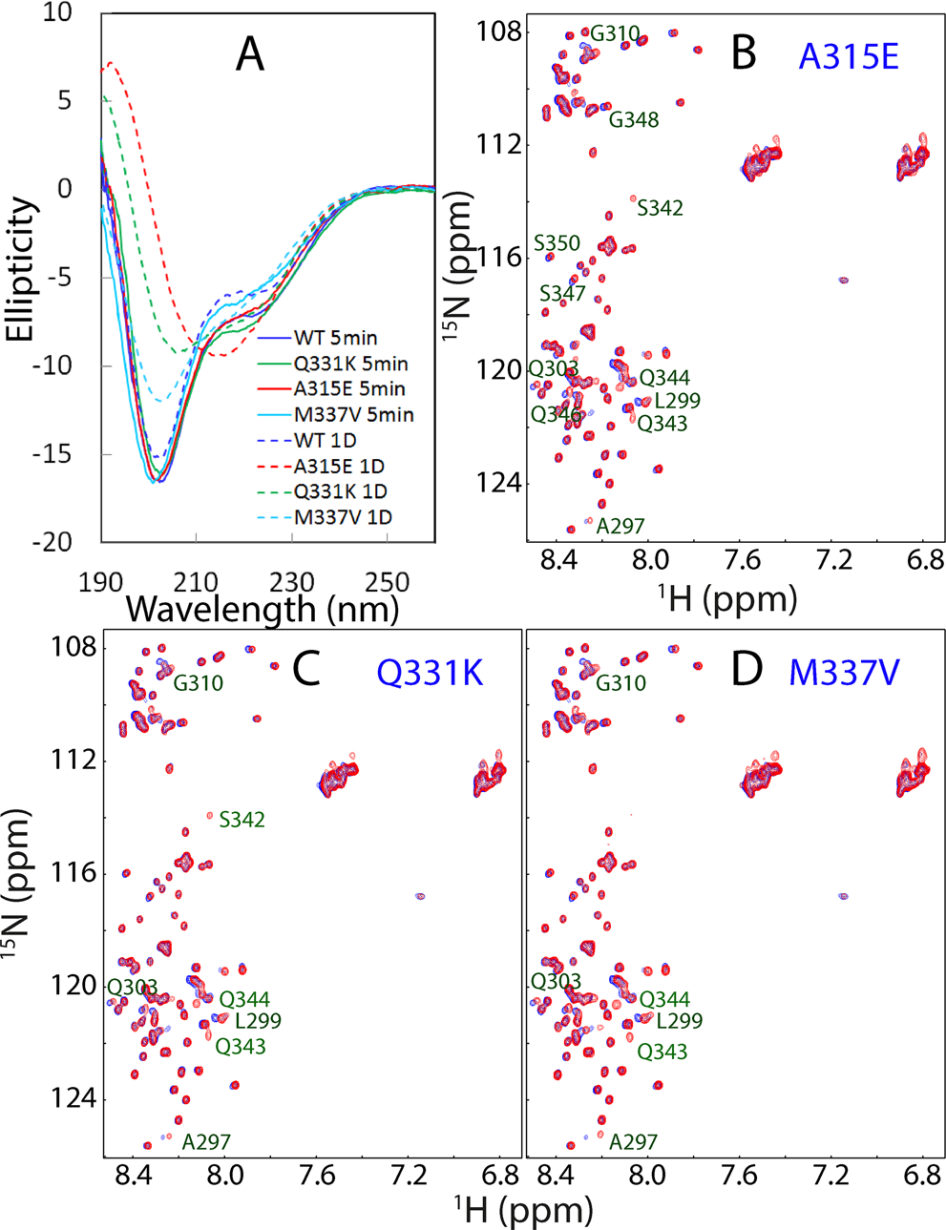
Conformations of the wild type and three mutants in bicelle. (A) Far-UV CD spectra of the wild-type and three mutant domains acquired at 25°C in the presence of DMPC/DHPC bicelle at a ratio of 1:200 after 5 min and 1 d. Superimposition of HSQC spectra acquired at 25°C in the presence of the DMPC/DHPC bicelle at a ratio of 1:200 for the wild type (blue) and mutants (red) for A315E (B), Q331K (C) and M337V (D). The mutant residues with HSQC peaks significantly shifted from those of the corresponding wild-type residues are labeled.

Subsequently, we titrated the wild type and mutants with the DMPC/DHPC bicelle which has relatively large and flat surface, thus better mimicking the bilayer membrane. Interestingly, the wild type and mutants also have very similar CD spectra 5 min after the addition of the bicelle at a ratio of 1:200 (Fig 10A). Furthermore, immediately after adding DMPC/DHPC bicelle, the residues of three mutants also have their HSQC peaks largely superimposable to those of the wild type (Fig 10B–10D).

Strikingly, however, 1 d after embedded in DMPC/DHPC bicelle, the CD spectra of the wild type showed no significant change, whereas those of three mutants underwent very large changes (Fig 10A). In particular, after 1 d, A315E had a CD spectrum typical of the amyloid oligomer. On the other hand, if compared their HSQC spectra, after 1 d, no new peak or significant peak shifts were observed. Instead, the peak intensity for three mutants reduced significantly. After 1 wk, visible aggregates were observed for three mutants but not the wild type. This implies that the changes of the CD spectra observed on three mutants (Fig 10A) are largely resulting from the self-association of three mutant proteins in the bicelle.

As well-established [71–75,79], the DPC micelle has very high curvature as well as small volume which may stably accommodate only one molecule with the size of the TDP-43 prion-like domain. As such, the self-association/aggregation is anticipated to be largely inhibited in DPC micelle. By contrast, the DMPC/DHPC bicelle has the relatively large and flat membrane surface, and thus can host more than one protein molecules in one bicelle. As a consequence, the local concentration of the proteins will be significantly increased, and their self-association might be significantly enhanced. This explains why the self-association is very minor even for the three ALS-causing mutants in the DPC micelle, but become much faster in DMPC/DMHC bicelle. Indeed, nature has used this strategy to increase the local protein concentration for effective clotting by anchoring the gamma-carboxyglutamic acid-rich (GLA) domain of human prothrombin onto membrane surfaces [84,85].

## Discussion

Our views about ALS pathogenesis are undergoing a paradigm shift triggered by the discovery that the pathogenic inclusions of TDP-43 presented in ~97% ALS and ~45% FTD patients regardless of being familial or sporadic [5]. Furthermore, TDP-43 inclusions have also been observed in many other neurodegenerative diseases and recently it was clinically revealed that only the Alzheimer patients with TDP-43 inclusions have significant cognitive impairment [15]. On the other hand, dynamic assembly into functional oligomers mainly mediated by the prion-like domain has been extensively demonstrated to be essential for the physiological functions of TDP-43 and consequently it was recently proposed that ALS pathogenesis may be initiated by a transition from the reversible assembly to irreversible aggregation under pathological conditions. Indeed, the wild-type TDP-43 itself is intrinsically aggregation-prone as well as toxic but the ALS-causing mutations appear to significantly exaggerate it.

In the present study, as facilitated by our previous discovery [33,38,46,47,62,63,68], for the first time, to the best of our knowledge, we have successfully determined the conformations and dynamics of the full-length TDP-43 prion-like domain at atomic-resolution by NMR spectroscopy. In aqueous solution, the TDP-43 prion-like domain is intrinsically disordered, which only contains some nascent secondary structures with highly-unrestricted backbone motions on ps-ns time scale. Unexpectedly, despite being mostly monomeric and disordered, the TDP-43 prion-like domain in water at pH 4.0 contains a large number of inter-molecular hydrogen bonds between side chain and backbone atoms, as particularly evidenced by the manifestation of the intrinsic visible fluorescence and low temperature coefficients of backbone amides. Furthermore, at neutral pH, the monomeric prion-like domain starts to assemble into the oligomer with a transition from the disordered to β-sheet rich conformations as reported by CD and three fluorescence probes, which has amyloid-like fibrillar structures as imaged by EM (Fig 4A). Mechanistically, the assembly appears to be initiated by the liberation of the QNS side chains from intra-molecular hydrogen bonding to form inter-molecular “hydrogen bonds/steric zippers” as previously proposed [22,67,70]. Nevertheless, at high protein concentrations, even the wild type would become precipitated rapidly with the formation of white aggregates of amorphous structures (Fig 4E).

By a sharp contrast, despite having average conformations highly similar to that of the wild type, all three ALS-causing point mutants namely A315E, Q331K and M337V gain the ability to transform into well-formed amyloid oligomers as particularly indicated by CD and intrinsic visible fluorescence spectra, which are very different from those of the wild type. Three mutants also form amyloid fibrils (Fig 4B–4D), with the morphology similar to that by the wild type (Fig 4A), as shown by EM, although their secondary structures might have some difference from those of the wild type. Previously, it has been revealed that the prion-like domains alone of FUS and TDP-43 are sufficient to bind nucleic acids to initiate the functional assembly [30,49]. Here we confirm this discovery and further show that the interaction with ssDNA facilitates the assembly of the wild type into the hydrogel. By a dramatic contrast, at the same ratios (protein:ssDNA), the interactions with ssDNA trigger immediate and irreversible precipitation with white aggregates for three mutants. The changes observed here in the self-assembly and interaction with nucleic acids may significantly reduce the functional capacity of the ALS-causing mutants, thus contributing to “loss of the functions” in ALS pathogenesis.

So how could we rationalize such radical effects of the ALS-causing point mutations on the TDP-43 prion-like domain, which is in fact highly disordered? Previously it has been well established that the intrinsically disordered proteins (IDPs), such as the TDP-43 prion-like domain, are in a dynamic equilibrium between different sets of conformations. So the monomeric state is characteristic of a relatively flat but rugged energy landscape with numerous local energy minima separated by low energetic barriers [22–24,30,49,50,87–89]. As a consequence, IDPs are, in fact, predicted to have very high specificity in binding as well as self-assembly. Indeed, it has been previously revealed that yeast uses the conformational diversity of its prion protein Sup35 to dictate its seeding specificity [22–25]. Therefore, the prion-like domains, such as in yeast Sup35 and human TDP-43, appear to represent a subgroup of the intrinsically disordered proteins that utilizes extremely high specificity in the assembly to achieve their functions.

**Fig 11:**
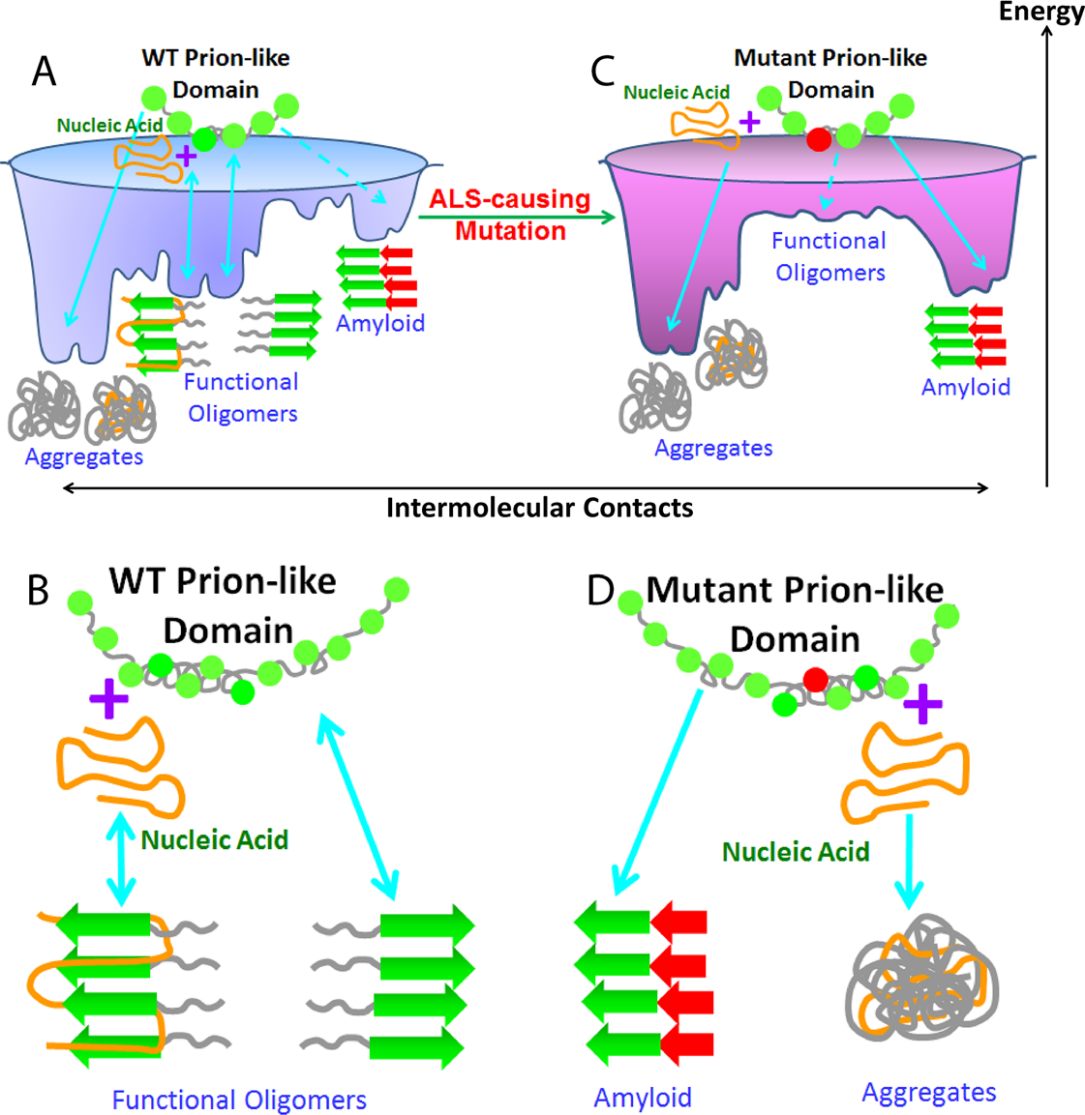
ALS-causing point mutations remodel the energy landscape of the self-assembly of the TDP-43 prion-like domain. (A) Energy landscape of the self-association of the wild-type prion-like domain; and (B) its assembly of the dynamic oligomers by self-association or interacting with nucleic acids, which is characteristic of the presence of a small portion of disordered regions. (C) Energy landscape of the self-association of the ALS-causing mutants; and (D) formation of the amyloid oligomers by self-association or aggregates upon interacting with nucleic acids. The dashed arrows are used to indicate the pathways which are likely to be inaccessible.

The wild-type TDP-43 prion-like domain appears to have an energy landscape to allow the self-assembly of reversible and functional oligomers only under limited conditions (Fig 11A and 11B). For instance, if upon pathological overexpression, the TDP-43 concentration is too high, the prion-like domain may directly jump to form irreversible aggregates with amorphous structures (Fig 4E), which were most frequently identified in the neuronal inclusions. Alternatively, if the dissociation of the functional oligomers is inhibited due to a long stress time or by other pathological conditions, they may lose the reversibility, thus transforming into irreversible aggregates or amyloid fibrils (Fig 4A), which was also detected in patients’ brain tissues [57]. As the assembly is extremely specific, an ALS-causing point mutation even like M337V which only has very minor change of the side chain is sufficient to remodel the energy landscape, at least partly by perturbing the hydrogen network, to more favor the formation of irreversible aggregates or/and amyloid oligomers (Fig 11C and 11D). In the future, more ALS-causing mutants should be studied to better understand the molecular mechanism by which the mutations globally remodel the energy landscape.

On the other hand, only aggregation of TDP-43 alone appears insufficient to cause “gain of neurotoxicity” because it was recently shown that the in vivo aggregation by the sequence 331–369 repeated 12 times in fact prevented TDP-43 neurotoxicity [31]. Here, our identification of a membrane-interacting subdomain over residues Met311-Gln343 may reconcile the paradox that on one hand, the TDP-43 prion-like domain is highly neurotoxic. On the other hand, its aggregation could be protective. Previously, all aggregation-prone proteins causing human diseases have been demonstrated to contain membrane-interacting domains/fragments. Therefore, here we propose that the coupled capacity of the membrane-interaction and aggregation might critically account for the high neurotoxicity of TDP-43. Indeed, previous studies have extensively demonstrated that the membrane-interacting regions, particularly that forming the Ω-loop, are absolutely indispensable for the neurotoxicity of the TDP-43 fragments [39,43,44], and its absence in fact converted the aggregation to become protective [31,45]. Here we also found that upon being associated onto the large surface of the DMPC/DHPC bicelle, the self-association of three ALS-causing mutants has been significantly sped up as compared to that in solution. Therefore, the membrane disruption by TDP-43 inclusion may also follow similar mechanisms previously established for other disease-causing proteins such as amyloid-β and IAPP(20–29) peptides. Briefly, under pathological conditions, the wild-type TDP-43 or its mutant molecules associated onto the membranes may further form large aggregates/inclusions to trigger nonspecific fragmentation of the lipid membrane [71–75]. In the future, it is also of interest to explore whether the membrane-interaction is essential for the physiological function of TDP-43. In fact, it has been realized that for the prion-like domains of FUS and TDP-43, the protein concentrations required for the specific assembly in vitro are much higher than the physiological concentrations in cells. So, it has been proposed that the additional binding with nucleic acids by RNA binding motifs may act to increase the local concentration to allow the specific assembly [30,48,49]. Here we speculate that the dynamic association with membranes might offer an alternative mechanism to effectively increase the local concentration as well as properly align the individual prion-like domain for the specific assembly, as found with the human GLA domain [84,85]. Nevertheless, if this physiological membrane-association is exaggerated by pathological factors, TDP-43 might become significantly aggregated in membranes, leading to the loss of its physiological functions as well as gain of neurotoxicity. As such, to decouple the aggregation and membrane interaction may represent a promising therapeutic strategy to treat neurodegenerative diseases.

## Methods

### Preparation of Recombinant Proteins

The DNA encoding the full-length prion-like domain over residues Lys363-Met414 was amplified by PCR reactions from the full-length TDP-43 gene and subsequently cloned into a modified vector pET28a with six His residues at C-terminus. Three ALS-causing mutations, A315E, Q331K, and M337V, were introduced into the TDP-43 prion-like domain by use of the QuikChange Site-Directed Mutagenesis Kit (Stratagene, La Jolla, CA, United States) as previously described [62,68]. The expression vectors were subsequently transformed into and overexpressed in *Escherichia coli* BL21 (DE3) cells (Novagen). The recombinant wild-type and mutant proteins were all found in inclusion body. As a result, the pellets were first dissolved in a phosphate buffer (pH 8.5) containing 8 M urea and subsequently purified by a Ni^2+^-affinity column (Novagen) under denaturing conditions in the presence of 8 M urea. The fractions containing the recombinant proteins were acidified by adding 10% acetic acid and subsequently purified by reverse-phase (RP) HPLC on a C4 column eluted by water-acetonitrile solvent system. The HPLC elutions containing pure recombinant proteins were lyophilized.

The generation of the isotope-labeled proteins for NMR studies followed a similar procedure except that the bacteria were grown in M9 medium with the addition of (^15^NH_4_)_2_SO_4_ for ^15^N labeling and (^15^NH_4_)_2_SO_4_/[^13^C]-glucose for double labelling [28,33,48,62,68]. The purity of the recombinant proteins was checked by SDS–PAGE gels and their molecular weights were verified by a Voyager STR matrix-assisted laser desorption ionization time-of-flight-mass spectrometer (Applied Biosystems). The concentration of protein samples was determined by the UV spectroscopic method in the presence of 8 M urea. Briefly, under the denaturing condition, the extinct coefficient at 280 nm of a protein can be calculated by adding up the contribution of Trp, Tyr, and Cys residues [90].

### CD and NMR Experiments

All CD experiments were performed on a Jasco J-810 spectropolarimeter equipped with a thermal controller using 1-mm path length cuvettes. Data from five independent scans were added and averaged. The wild-type and mutant TDP-43 prion-like domain samples were prepared at a protein concentration of 20 μM either in Milli-Q water (pH 4.0), or 1 mM phosphate at pH 5.0, 6.0 and 6.8 respectively. For characterizing the interactions of (TG)_6_ ssDNA we previously used for the TDP-43 N-domain [33] with the wild-type and three mutant prion-like domains, the protein samples at 20 μM were prepared in 1 mM phosphate buffer at pH 5.0 while the ssDNA was dissolved in the same buffer. The CD spectra of the proteins at different ratios of protein:ssDNA were obtained by calibrating the dilution factor as well as subtracting the CD contribution of ssDNA at the corresponding ratios. CD spectra were further analyzed to estimate secondary structure contents as we previously conducted [33,63] by CDPro software package (lamar.colostate.edu/∼sreeram/CDPro/main.html).

In the present study, two membrane-mimetic systems, namely the DPC micelle and DMPC/DHPC bicelle, were used to identify the membrane-interacting regions of the TDP-43 prion-like domains. In aqueous solution, dodecylphosphocholine (DPC) self-assembles into the micelle structure typically containing ~50–100 molecules. The large bicelle to better mimic bilayer membrane was prepared by mixing up dimyristoylphosphatidylcholine (DMPC) and dihexanoylphophatidylcholine (DHPC) at a q value of 4 as previously described [79]. At this ratio, the disk-shaped bicelle with a diameter of ~460 Å is formed in which DMPC constitutes a bilayered section surrounded by a rim of DHPC [79,91].

All NMR experiments were acquired on an 800 MHz Bruker Avance spectrometer equipped with pulse field gradient units as described previously [33,78]. For characterizing the conformations in aqueous solutions, a pair of triple-resonance experiments HNCACB, CBCA(CO)NH were collected for the sequential assignment on a ^15^N-/^13^C-double labelled sample of 500 μM, while ^15^N-edited HSQC-TOCSY and HSQC-NOESY were collected on a ^15^N-labelled sample at a protein concentration of 500 μM. For achieving assignments in DPC micelle, triple-resonance experiments HNCACB, CBCA(CO)NH and HCCH-TOCSY were acquired on ^15^N-/^13^C-double labelled samples at a protein concentration of 500 μM in the DPC micelle (H-DPC) at 100 mM. For obtaining NOE connectivities, ^15^N-edited HSQC-TOCSY and HSQC-NOESY were collected on a ^15^N-labelled sample at a protein concentration of 500 μM in DPC micelles at 100 mM. NMR data were processed with NMRPipe [92] and analyzed with NMRView [93].

For assessing the backbone dynamics on the ps-ns time scale, {^1^H}-^15^N steady-state NOEs were obtained by recording spectra on the ^15^N-labeled sample at 500 μM in either aqueous solution or DPC micelle (100 mM), with and without ^1^H presaturation with duration of 3 s plus a relaxation delay of 6 s at 800 MHz. To assess conformational exchanges over μs-ms, ^15^N transverse relaxation dispersion experiments were acquired on the ^15^N-labeled sample at 500 μM in either aqueous solution or DPC micelle (100 mM), on a Bruker Avance 800 spectrometer with a constant time delay (T_CP_ = 50 ms) and a series of CPMG frequencies, ranging from 40 Hz, 80 Hz, 120 Hz (x3), 160 Hz, 200 Hz, 240 Hz, 320 Hz, 400 Hz, 480 Hz, 560 Hz, 640 Hz, 720 Hz, 800 Hz, and 960 Hz (×3 indicates repetition) as we previously performed [68,78].

To probe the accessibility of the prion-like domain residues in DPC micelle [78], HSQC spectra at 100 μM in the presence of 20 mM DPC were acquired by gradual addition to 10 mM of gadodiamide (gadolinium(III) 5,8-bis(carboxylatomethyl)-2-[2-(methylamino)-2-oxoethyl]-10-oxo-2,5,8,11-tetraazadodecane-1-carboxylate hydrate).

### Fluorescence Spectral Measurements

All fluorescence spectra were measured at 25°C with a RF-5301 PC spectrophotometer (Shimadzu, Japan) as previously established [51–56], at different time points of the incubations of the wild-type and three mutants at a protein concentration of 40 μM in 1 mM phosphate buffer (pH 6.8). The rectangular fluorescence quartz cuvette has the pathlength dimension of 10 x 10 mm and the general settings are: PMT at low sensitivity and scan speed of medium speed (200 nm/min). For the intrinsic UV fluorescence, the emission spectra were measured with the excitation wavelength at 280 nm and slit widths: excitation at 5 nm and emission at 10 nm. For the intrinsic visible fluorescence, the emission spectra were measured with the excitation wavelength at 375 nm and slit widths: excitation at 20 nm and emission at 10 nm.

For Thioflavin-T (ThT) binding assay, a 2 mM ThT stock solution was prepared by dissolving ThT in milli-Q water and filtered through a 0.22 μm Millipore filter. The fresh working solution was prepared by diluting the stock solution into 1 mM phospate buffer (pH 6.8) to reach a final ThT concentration of 50 μM. A 10 μL aliquot of each incubation solution, or 10 μL aliquot of the incubation buffer (1 mM phosphate at pH 6.8) as the control, was mixed with 130 μL of the ThT working solution in the dark for 10 min. The fluorescence emission spectra were acquired for three repeats with the excitation wavelength at 442 nm and slit widths: excitation at 5 nm and emission at 10 nm.

### Electron Microscopy Imaging

Incubation samples of the wild type and three mutants at 40 μM were imaged at one and two weeks of the incubation in 1 mM phosphate buffer (pH 6.8), by a TEM microscope (Jeol Jem 2010f Hrtem, Japan) operating at an accelerating voltage of 200 kV. The aggregates of the wild type were prepared immediately before EM imaging, by diluting the stock protein sample in Milli-Q water (pH 4.0) into 1 mM phosphate buffer to reach a concentration of 200 μM (pH 6.8).

For EM imaging, a 5 μl aliquot of the incubation or aggregate solutions was placed onto the Cu grids (coated with carbon film; 150 mesh; 3 mm in diameter) and negatively stained with 5 μl of 2% neutral, phosphotungstic acid (PTA). This aliquot was allowed to settle on Cu grid for 30 s before the excess fluid was drained away. The Cu grid was later air-dried for another 15 mins before being imaged.

### Structure Modelling

Structure calculation was conducted on residues Met307-Ser347 with a large amount of NOEs in DPC micelle. Backbone dihedral angles were generated with TALOS+ by inputting backbone ^1^H, ^15^N and ^13^C chemical shifts. NOE-based distance constraints were extracted only from ^15^N-edited NOESY spectrum as the side-chain ^13^C NMR resonances are too broad to assign NOE connectivities as we previously observed [38,78]. The NMR structures in the DPC were calculated with the input of distance and dihedral angle constraints by CYANA. Six lowest target-function CYANA structures with no NOE violation >0.4 Å and no dihedral angle violation >4 degrees were selected for further refinement by use of GROMACS version 4.5.3 in Amber99sb-ildn force field, with dihedral angle and distance restraints converted and incorporated into the topology file.

The structure coordinate of the TDP-43 membrane-interacting subdomain over residues Met307-Ser347 in DPC micelle has been deposited in PDB with ID of 2N2C and the associated NMR data were also deposited in BMRB with ID of 25595.

## Supporting Information

S1 Data NMR data for the TDP-43 prion-like domain in aqueous solution. (XLSX)

S2 Data NMR data for the TDP-43 prion-like domain in DPC. (XLSX)

S1 Fig CD characterization of the wild-type TDP-43 prion-like domain at different pH values and temperatures. Far-UV CD spectra of the wild-type prion-like domain at temperatures from 20 to 90°C in Milli-Q water at pH 4.0 (A); in 1 mM phosphate buffer at pH 5.0 (B); and in 1 mM phosphate buffer at pH 6.0 (C).
(TIF)

S2 Fig CD and NMR characterization of three mutants in aqueous solution. (A) Mutation sites indicated in the membrane-embedded structure of the subdomain Met307-Ser347. (B) Far-UV CD spectra acquired at 25°C of the wild type and three mutants in 1 mM phosphate buffer at pH 5.0. Superimposition of HSQC spectra acquired at 25°C in 1 mM phosphate buffer at pH 5.0 for the wild-type prion-like domain (blue) and mutants (red) for A315E (C), Q331K (D) and M337V (E). The mutant residues with HSQC peaks shifted from those of the corresponding wild-type residues are labeled.
(TIF)

S3 Fig Fluorescence characterization of the self-association. Emission spectra of the intrinsic UV fluorescence for the wild type (A), A315E (B), Q331K (C) and M337V (D) in water at pH 4.0, and in 1 mM phosphate buffer at pH 6.8 at different time points of the incubation. The wavelengths of the emission maxima are labeled for the spectra of the samples in water (pH 4.0), 5 min, 1 d and 8 d after dilution into 1 mM phosphate buffer at pH 6.8. Emission spectra of the ThT-binding induced fluorescence for the wild type (E), A315E (F), Q331K (G), and M337V (H) in water at pH 4.0, and in 1 mM phosphate buffer (pH 6.8) at different time points of the incubation, which have the typical emission maximum at ~486 nm.
(TIF)

S4 Fig Electron microscope imaging. EM images of the samples incubated for 2 week for the wild type (A), A315E (B), Q331K (C), or M337V (D). Upper are images of lower magnification (scale bar of 1 μM) while lower are of higher magnification (scale bar of 200 nm).
(TIF)

S5 Fig Hydrophobicity of the TDP-43 prion-like domain. (A) Kyte & Doolittle hydrophobic scale of the prion-like domain. The green arrow is used to indicate the regions with positive scale. (B) Secondary structure score of the prion-like domain in aqueous solution obtained by analyzing chemical shifts with the SSP program. A score of +1 is for the well-formed helix while a score of -1 for the well-formed extended strand.
(TIF)

S6 Fig Residue-specific temperature coefficients of three mutants. Residue-specific temperature coefficients of the wild-type (blue) and mutant (red) residues in Milli-Q water at pH 4.0, in 1 mM phosphate buffer at pH 5.0, and in 1 mM phosphate buffer at pH 6.0.
(TIF)

S7 Fig CD characterization of the interactions with ssDNA. Far-UV CD spectra acquired at 25°C in 1 mM phosphate buffer at pH 5.0 in the presence of ssDNA at different ratios for the wild-type (A), A315E (B), Q331K (C), and M337V (D).
(TIF)

S8 Fig HSQC characterization of the interaction of ssDNA with the wild type. Superimposition of HSQC spectra acquired at 25°C in 1 mM phosphate buffer at pH 5.0 for the wild-type prion-like domain at a concentration of 40 μM, in the presence of ssDNA at molar ratios (protein:ssDNA) of 1:0 (blue), 1:0.5 (red), and 1:1 (green). The residues with their HSQC peaks detectable at the molar ratio of 1:0.5 are labeled. The asterisks are used to indicate residues which could not be assigned due to the overlap and missing of the side chain peaks in the triple-resonance NMR spectra.
(TIF)

S9 Fig Conformations of the wild-type prion-like domain in DPC micelle at different pH. (A) Far-UV CD spectra of the prion-like domain acquired at 25°C in Milli-Q water at pH 4.0 (black), in the presence of DPC micelle at different pH. (B) Superimposition of HSQC spectra of the prion-like domain acquired at 25°C in the presence of DPC micelle at a ratio of 1:200 in Milli-Q water at pH 4.0 (blue), and in 1 mM phosphate buffer at pH 5.0 (red). (C) Superimposition of HSQC spectra of the prion-like domain acquired at 25°C in the presence of DPC micelle at a ratio of 1:200 in Milli-Q water at pH 4.0 (blue), and in 1 mM phosphate buffer at pH 6.8 (red).
(TIF)

S10 Fig Conformations of the wild type and three mutants in DPC micelle. (A) Far-UV CD spectra of the wild type and three mutants in 1 mM phosphate buffer at pH 5.0, acquired at 25°C in the presence of DPC micelle at a ratio of 1:200 after 5 min. Superimposition of HSQC spectra acquired at 25°C in the presence of DPC micelle at a ratio of 1:200 for the wild type (blue) and mutants (red) for A315E (B), Q331K (C), and M337V (D), in 1 mM phosphate buffer at pH 5.0. The mutant residues with HSQC peaks shifted from those of the corresponding wild-type residues are labeled.
(TIF)

S1 Table Statistics for six selected NMR structures of the TDP-43 membrane-interacting subdomain over Met307-Ser347 in DPC. (DOCX)

## Abbreviations

AD: Alzheimer’s disease
ALS: amyotrophic lateral sclerosis
CD: circular dichroism
DHPC: dihexanoylphophatidylcholine
DLB: dementia with Lewy bodies
DMPC: dimyristoylphosphatidylcholine
DPC: dodecylphosphocholine
EM: electron microscope
FTD: frontotemporal dementia
FTLD: frontotemporal lobar degeneration
FUS: fused in sarcoma
GLA: gamma-carboxyglutamic acid-rich
hNOEs: heteronuclear NOEs
hnRNP: heterogeneous nuclear ribonucleoprotein
HSQC: heteronuclear single quantum coherence spectroscopy
IDPs: intrinsically -disordered proteins
MSP: major sperm protein
NES: nuclear export signal
NLS: nuclear localization signal
NMR: nuclear magnetic resonance
NOE: nuclear Overhauser effect
P-bodies: processing bodies
RP: reverse-phase
RRM: RNA recognition motif
SGs: stress granule
SOD1: superoxide dismutase 1
TDP-43: TAR-DNA-binding protein-43
ThT: Thioflavin T
UV: ultraviolet
