# Supplementary figures and images for: ALS-Causing Mutations Significantly Perturb the Self-Assembly and Interaction with Nucleic Acid of the Intrinsically Disordered Prion-Like Domain of TDP-43
Source: PLoS Biol. 2016 Jan 6;14(1):e1002338. doi: 10.1371/journal.pbio.1002338 (PMC4703307; doi:10.1371/journal.pbio.1002338)

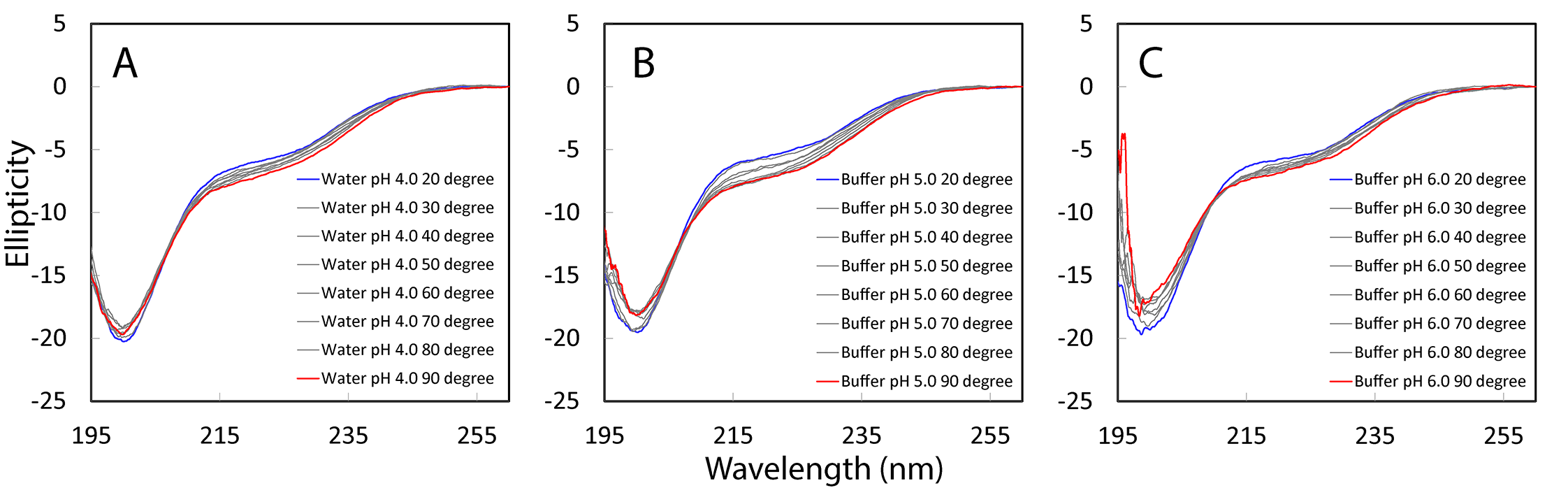

Supplement: S1 Fig — Far-UV CD spectra of the wild-type prion-like domain at temperatures from 20 to 90°C in Milli-Q water at pH 4.0 (A); in 1 mM phosphate buffer at pH 5.0 (B); and in 1 mM phosphate buffer at pH 6.0 (C). (TIF) [file pbio.1002338.s003.tif]

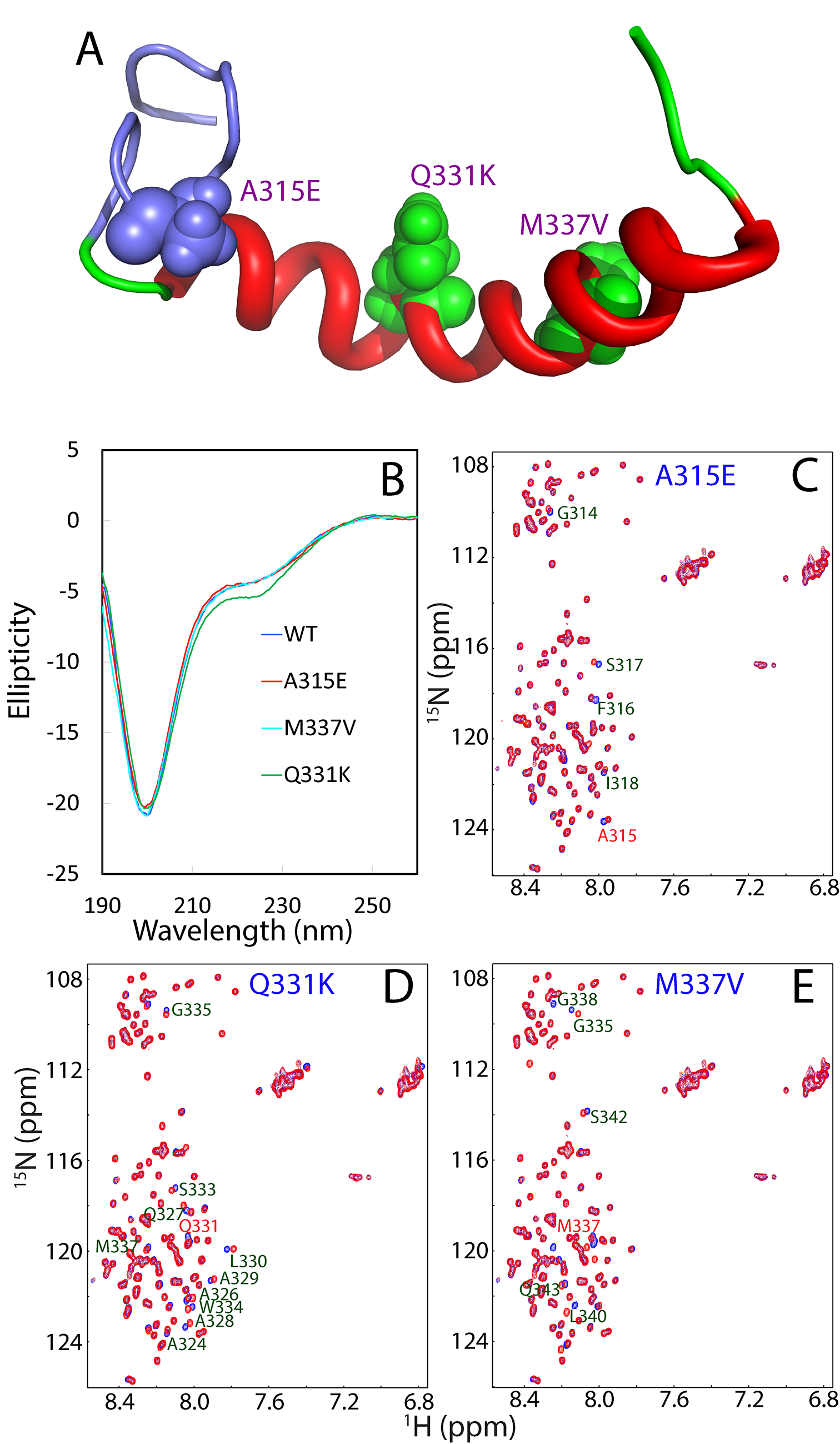

Supplement: S2 Fig — (A) Mutation sites indicated in the membrane-embedded structure of the subdomain Met307-Ser347. (B) Far-UV CD spectra acquired at 25°C of the wild type and three mutants in 1 mM phosphate buffer at pH 5.0. Superimposition of HSQC spectra acquired at 25°C in 1 mM phosphate buffer at pH 5.0 for the wild-type prion-like domain (blue) and mutants (red) for A315E (C), Q331K (D) and M337V (E). The mutant residues with HSQC peaks shifted from those of the corresponding wild-type residues are labeled. (TIF) [file pbio.1002338.s004.tif]

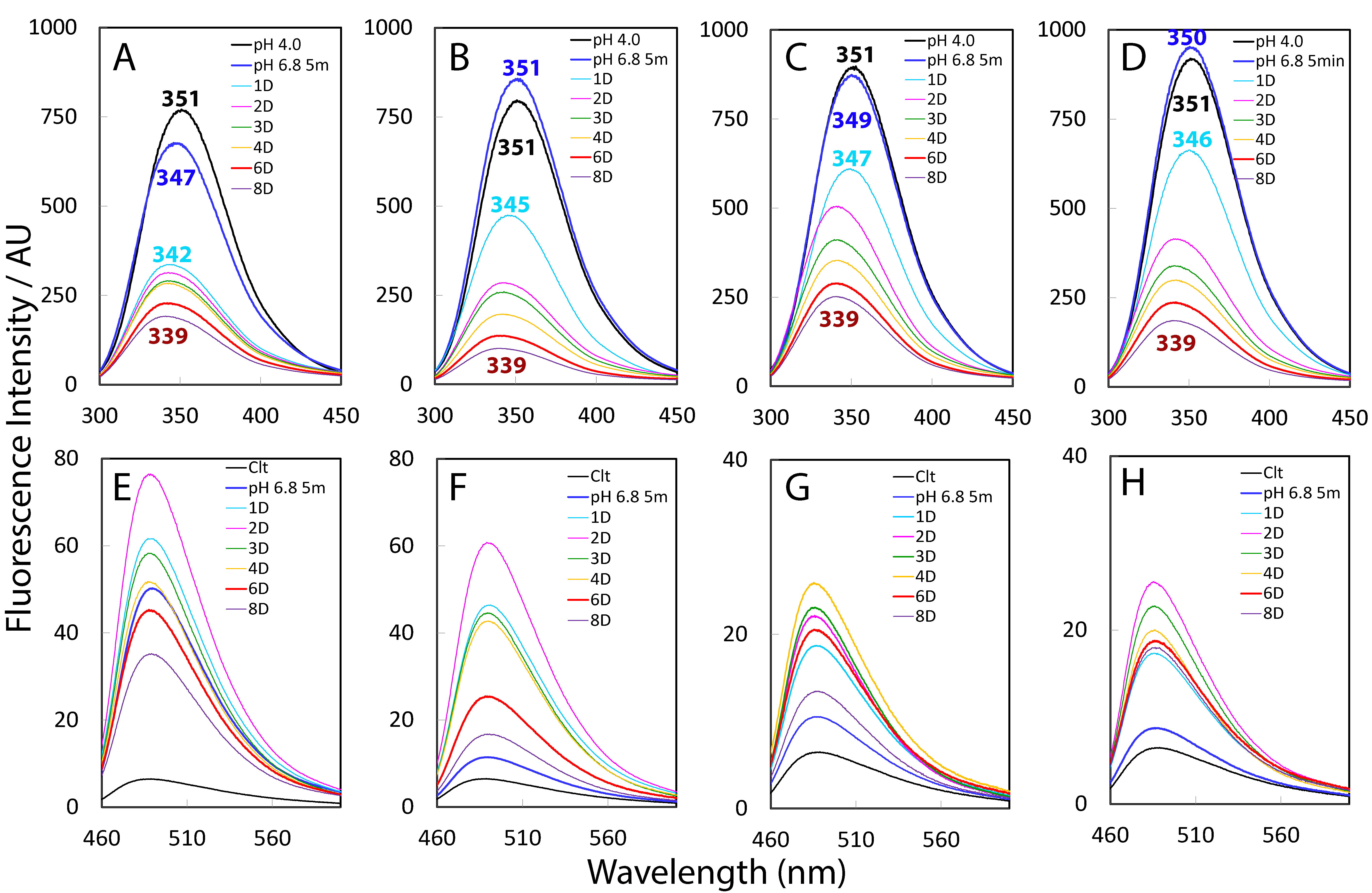

Supplement: S3 Fig — Emission spectra of the intrinsic UV fluorescence for the wild type (A), A315E (B), Q331K (C) and M337V (D) in water at pH 4.0, and in 1 mM phosphate buffer at pH 6.8 at different time points of the incubation. The wavelengths of the emission maxima are labeled for the spectra of the samples in water (pH 4.0), 5 min, 1 d and 8 d after dilution into 1 mM phosphate buffer at pH 6.8. Emission spectra of the ThT-binding induced fluorescence for the wild type (E), A315E (F), Q331K (G), and M337V (H) in water at pH 4.0, and in 1 mM phosphate buffer (pH 6.8) at different time points of the incubation, which have the typical emission maximum at ~486 nm. (TIF) [file pbio.1002338.s005.tif]

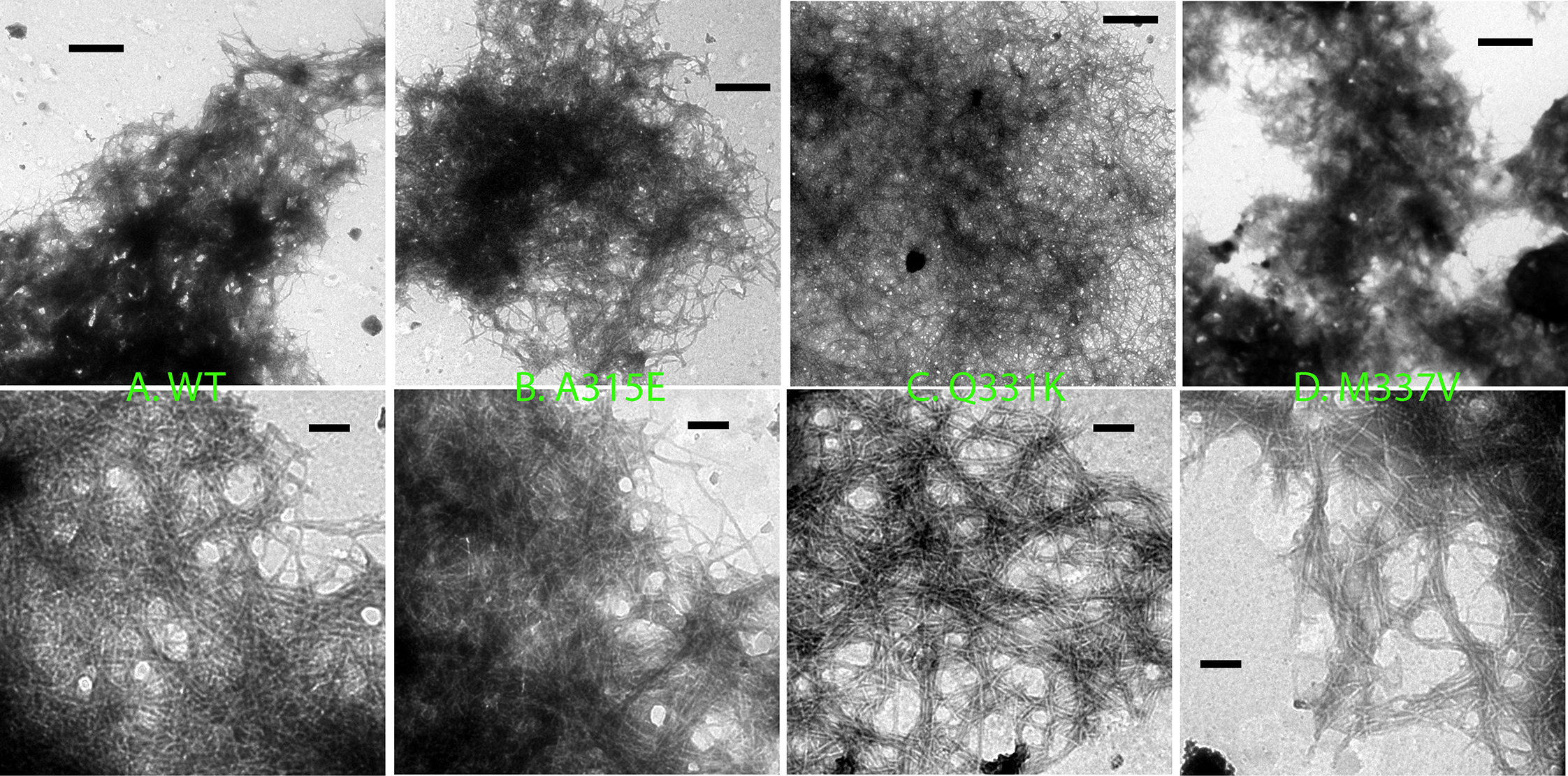

Supplement: S4 Fig — EM images of the samples incubated for 2 week for the wild type (A), A315E (B), Q331K (C), or M337V (D). Upper are images of lower magnification (scale bar of 1 μM) while lower are of higher magnification (scale bar of 200 nm). (TIF) [file pbio.1002338.s006.tif]

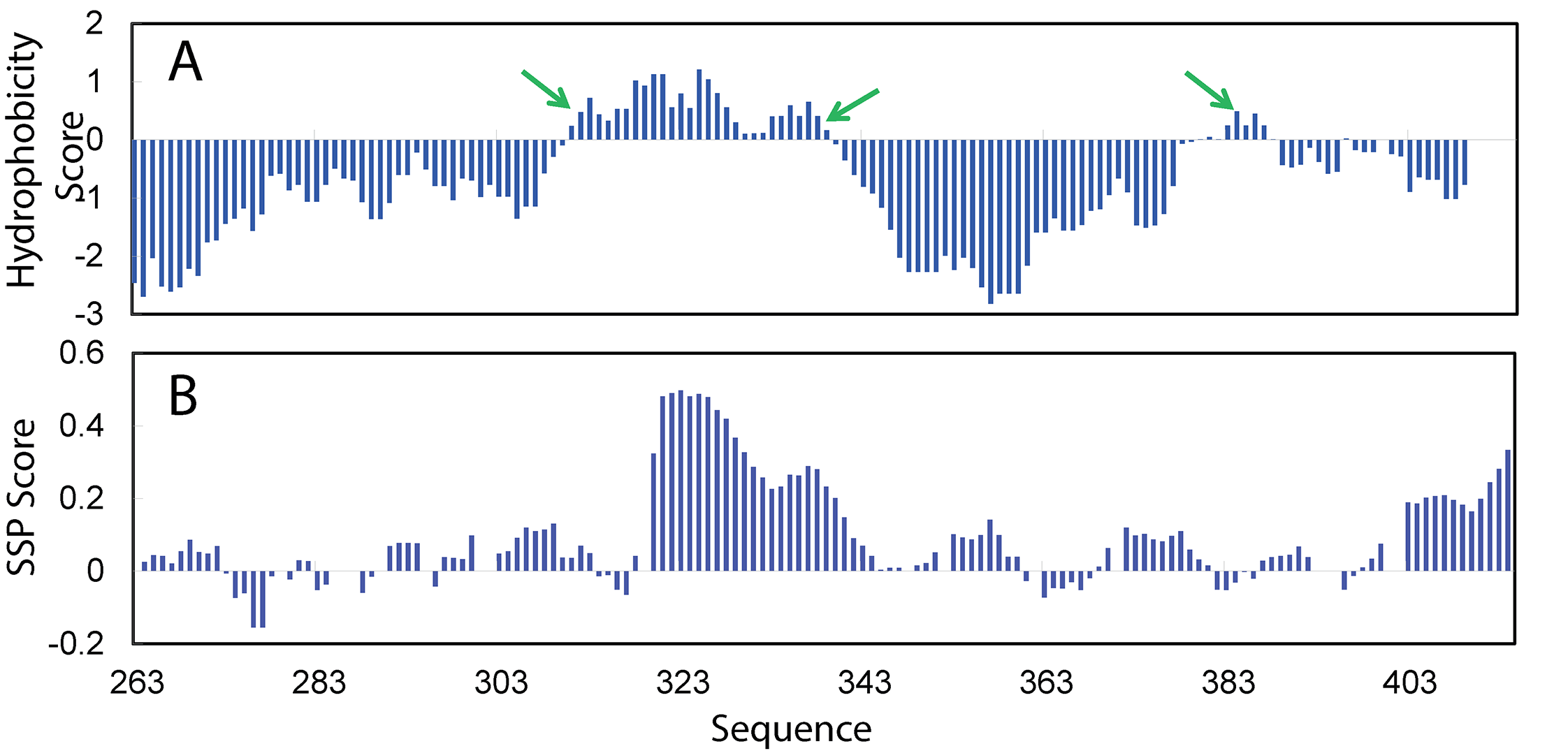

Supplement: S5 Fig — (A) Kyte & Doolittle hydrophobic scale of the prion-like domain. The green arrow is used to indicate the regions with positive scale. (B) Secondary structure score of the prion-like domain in aqueous solution obtained by analyzing chemical shifts with the SSP program. A score of +1 is for the well-formed helix while a score of -1 for the well-formed extended strand. (TIF) [file pbio.1002338.s007.tif]

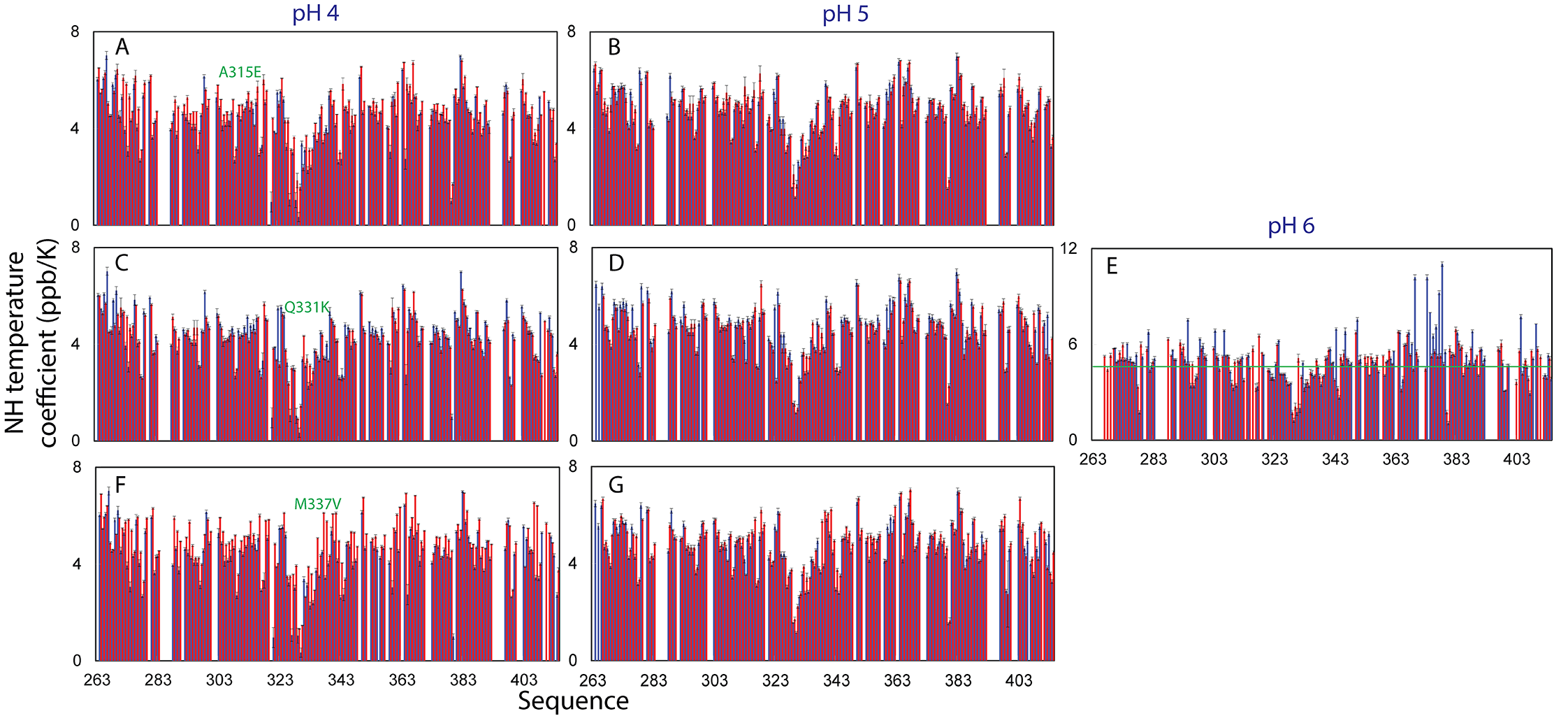

Supplement: S6 Fig — Residue-specific temperature coefficients of the wild-type (blue) and mutant (red) residues in Milli-Q water at pH 4.0, in 1 mM phosphate buffer at pH 5.0, and in 1 mM phosphate buffer at pH 6.0. (TIF) [file pbio.1002338.s008.tif]

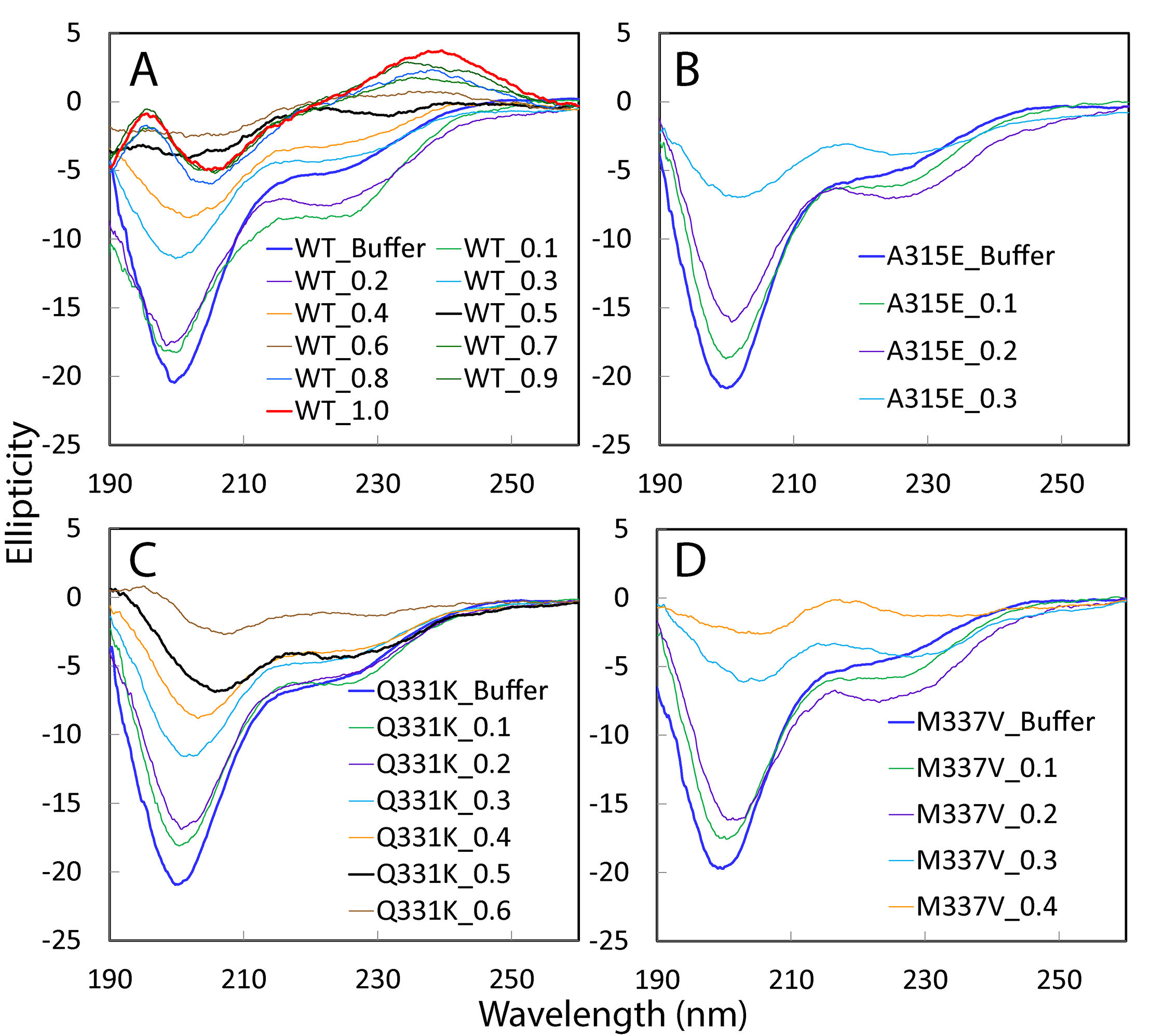

Supplement: S7 Fig — Far-UV CD spectra acquired at 25°C in 1 mM phosphate buffer at pH 5.0 in the presence of ssDNA at different ratios for the wild-type (A), A315E (B), Q331K (C), and M337V (D). (TIF) [file pbio.1002338.s009.tif]

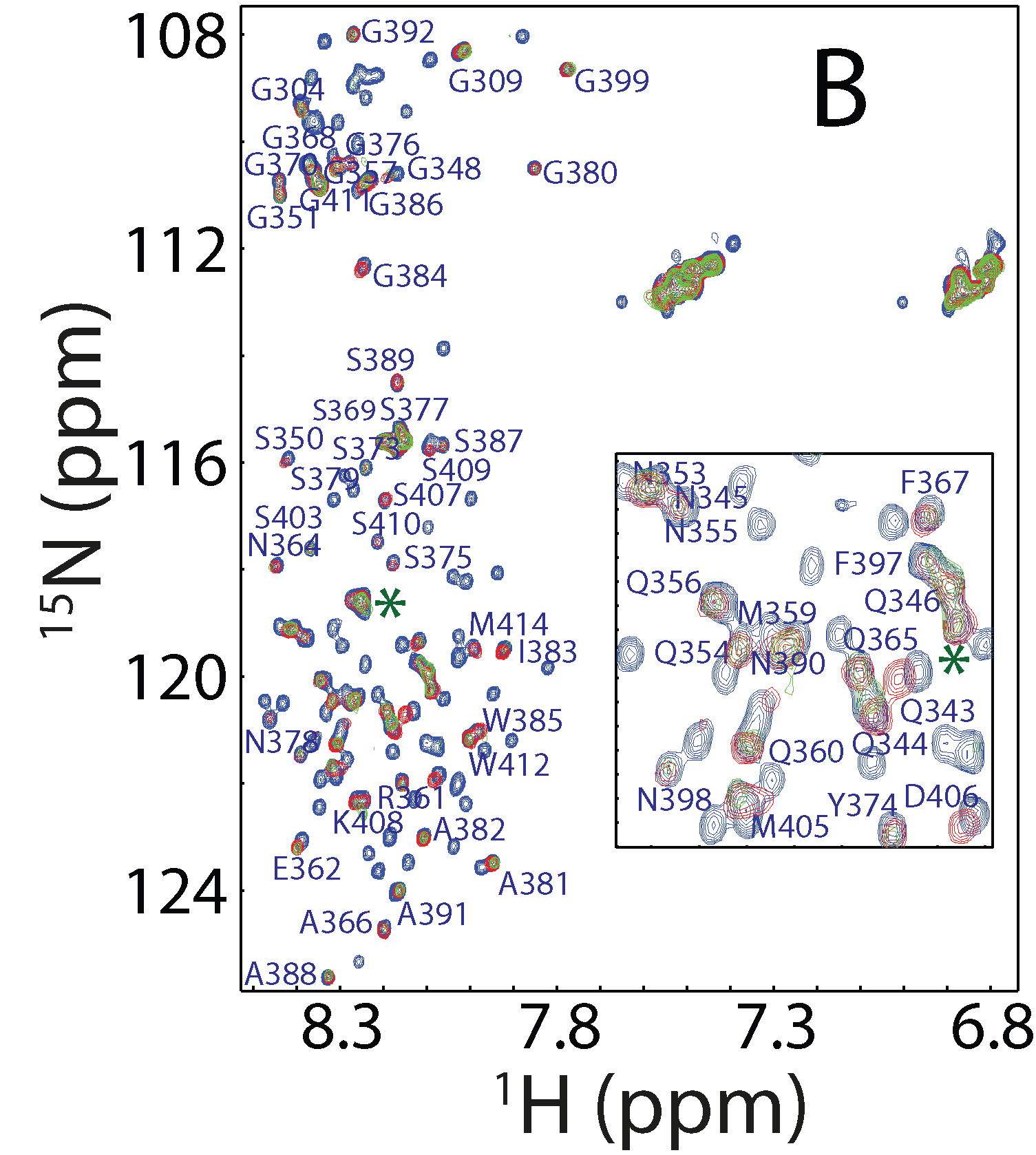

Supplement: S8 Fig — Superimposition of HSQC spectra acquired at 25°C in 1 mM phosphate buffer at pH 5.0 for the wild-type prion-like domain at a concentration of 40 μM, in the presence of ssDNA at molar ratios (protein:ssDNA) of 1:0 (blue), 1:0.5 (red), and 1:1 (green). The residues with their HSQC peaks detectable at the molar ratio of 1:0.5 are labeled. The asterisks are used to indicate residues which could not be assigned due to the overlap and missing of the side chain peaks in the triple-resonance NMR spectra. (TIF) [file pbio.1002338.s010.tif]

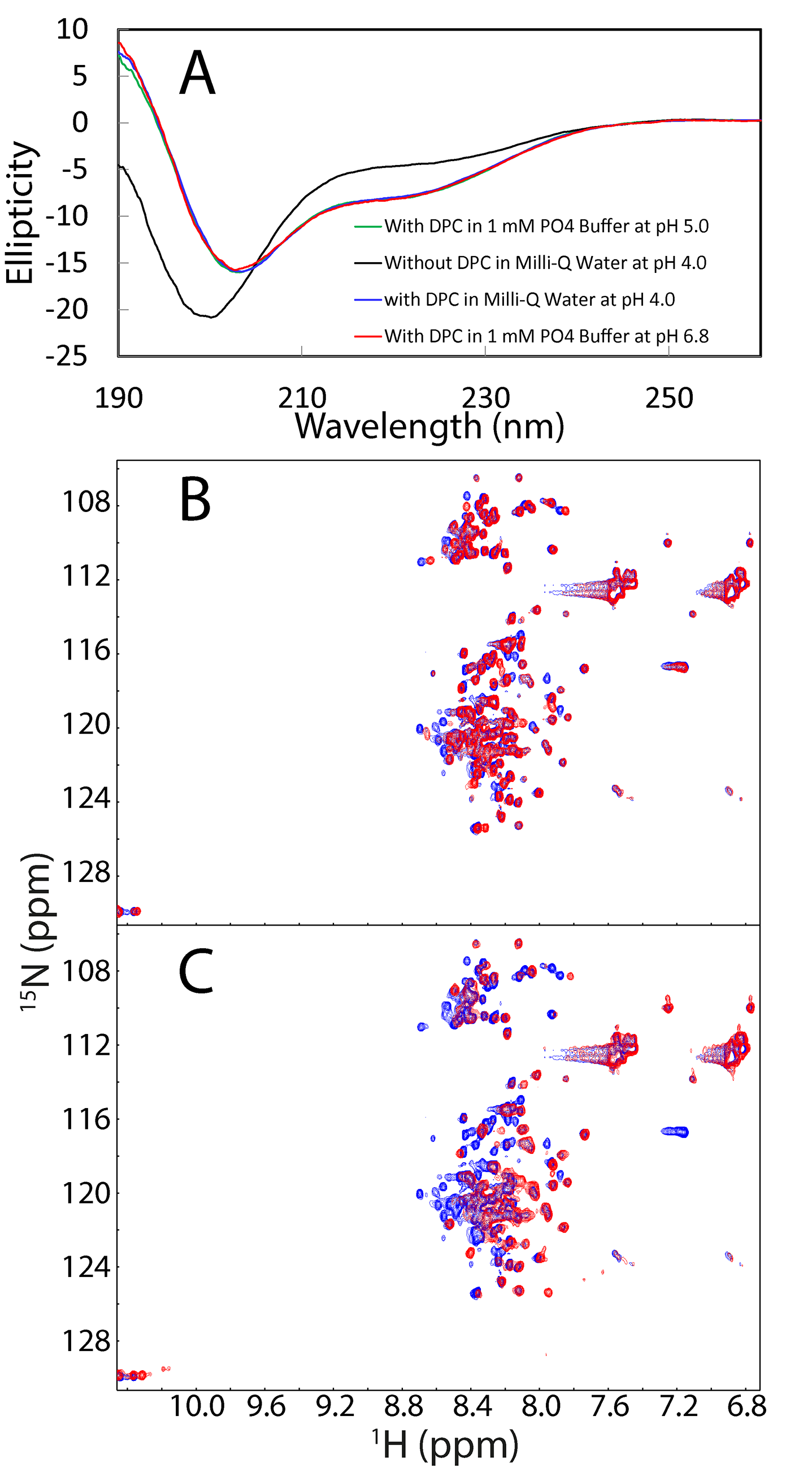

Supplement: S9 Fig — (A) Far-UV CD spectra of the prion-like domain acquired at 25°C in Milli-Q water at pH 4.0 (black), in the presence of DPC micelle at different pH. (B) Superimposition of HSQC spectra of the prion-like domain acquired at 25°C in the presence of DPC micelle at a ratio of 1:200 in Milli-Q water at pH 4.0 (blue), and in 1 mM phosphate buffer at pH 5.0 (red). (C) Superimposition of HSQC spectra of the prion-like domain acquired at 25°C in the presence of DPC micelle at a ratio of 1:200 in Milli-Q water at pH 4.0 (blue), and in 1 mM phosphate buffer at pH 6.8 (red). (TIF) [file pbio.1002338.s011.tif]

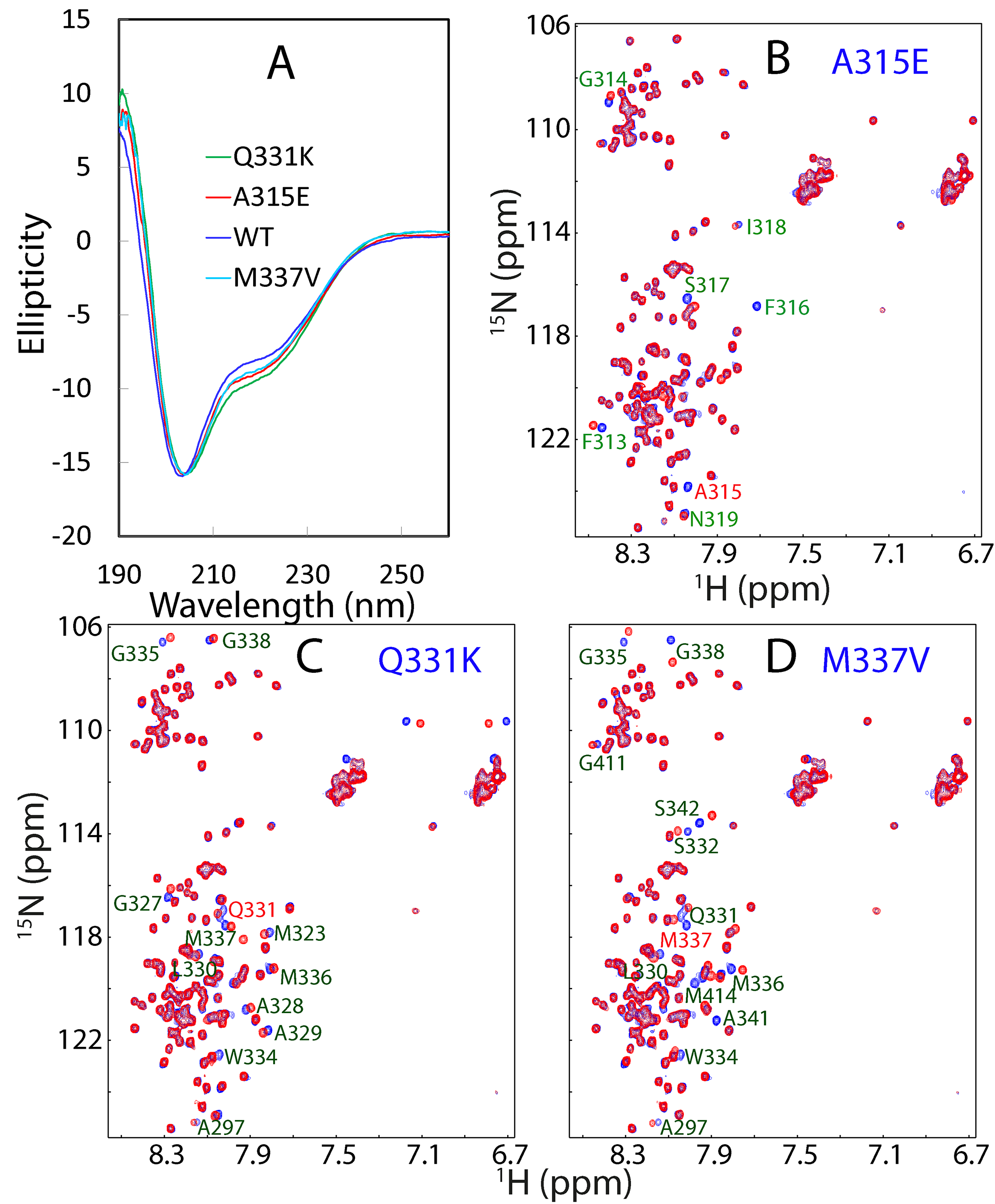

Supplement: S10 Fig — (A) Far-UV CD spectra of the wild type and three mutants in 1 mM phosphate buffer at pH 5.0, acquired at 25°C in the presence of DPC micelle at a ratio of 1:200 after 5 min. Superimposition of HSQC spectra acquired at 25°C in the presence of DPC micelle at a ratio of 1:200 for the wild type (blue) and mutants (red) for A315E (B), Q331K (C), and M337V (D), in 1 mM phosphate buffer at pH 5.0. The mutant residues with HSQC peaks shifted from those of the corresponding wild-type residues are labeled. (TIF) [file pbio.1002338.s012.tif]
