# Supplementary material for: ALS-Causing Mutations Significantly Perturb the Self-Assembly and Interaction with Nucleic Acid of the Intrinsically Disordered Prion-Like Domain of TDP-43
Source: PLoS Biol. 2016 Jan 6;14(1):e1002338. doi: 10.1371/journal.pbio.1002338 (PMC4703307; doi:10.1371/journal.pbio.1002338)
Supplement: S1 Table — (DOCX) [file pbio.1002338.s013.docx]

__________________________________________________________________________

**Experimental constraints for structure calculation**

NOE restraints

Total 328

Intra residue 111

Sequential 132

Medium 76

Long-range 9

Dihedral angle constraints

Total 46

Phi 23

Psi 23

Distance violation > 0.5 Å 0

Angle violation > 5 º 0

CYANA target function 6.3

**Deviations from ideal geometry**

Bond 0.020 ± 0.006 Å

Angles 1.995 ± 0.866 °

Improper 11.981 ± 5.279 °

**Ramachandran statistics (%)**

Most favored 78.1

Additionally allowed 18.8

Generously allowed 3.1

Disallowed 0

**Root mean square deviation for Ω-loop and helix (311-344) (Å)**

All atoms 1.11 ± 0.34

Heavy atoms 0.91 ± 0.25

Backbone atoms 0.35 ± 0.11
